# Reversal of T cell exhaustion enables cancer vaccine efficacy in *BRCA1*-deficient ovarian cancer

**DOI:** 10.1016/j.isci.2026.116587

**Published:** 2026-06-30

**Authors:** Laurent Beziaud, Aspram Minasyan, Cheryl L-L Chiang, Rania M. Soukarieh, Matilde M. Coppi, Raphaël Rovelli, Jonathan Thevenet, Stephanie Tissot, Denarda Dangaj-Laniti, Lana E. Kandalaft

**Affiliations:** 1 Department of Oncology, Lausanne University Hospital, Ludwig Institute for Cancer Research, Lausanne Branch, University of Lausanne (UNIL), Agora Cancer Research Center, Lausanne, Switzerland; 2 Center of Experimental Therapeutics, Department of Oncology, Lausanne University Hospital, Lausanne, Switzerland; 3 Department of Oncology, Lausanne University Hospital, Ludwig Institute for Cancer Research, Lausanne Branch, University of Lausanne (UNIL), Agora Cancer Research Center, Swiss Medical Network, Genolier Innovation Network, Genolier Clinic, Genolier, Switzerland

**Keywords:** ovarian cancer, *BRCA1* mutation, cancer vaccine, T cell exhaustion, PARP inhibition, immune checkpoint blockade

## Abstract

BRCA mutations in ovarian cancer (OC) are associated with increased tumor-infiltrating lymphocytes and inflammatory features, yet immunotherapy for OC has shown limited efficacy. Using the ID8 mouse model, we discovered that *Brca1* deficiency—but not *Brca2*—impairs the effectiveness of a dendritic cell-based cancer vaccine (OCDC). OCDC vaccine reduced tumor growth and improved survival in mice bearing *Trp53*^−/−^ and *Trp53*^−/−^*Brca2*^−/−^ tumors but was ineffective against *Trp53*^−/−^*Brca1*^−/−^ tumors. Transcriptomic analysis revealed metabolic and immunologic reprogramming in OCDC-responsive tumors, whereas *Brca1*^−/−^ tumors remained refractory. Vaccine inefficacy was associated with a pre-existing inflamed microenvironment, enriched in activated yet exhausted T cells, limiting further immune stimulation by vaccination. Combining OCDC with anti-VEGF and PARP inhibitors partially restored vaccine efficacy in *Brca1*^−/−^ tumors, while addition of anti-PD-1 further enhanced T cell function and promoted long-term survival. These findings highlight *BRCA1*’s role in cancer vaccine sensitivity and support rational combination immunotherapies for *BRCA1*-mutated OC.

## Highlights

• *Brca1* deficiency—but not *Brca2*— in ovarian cancer impairs effectiveness of cancer vaccine
• Vaccine-responsive tumors undergo metabolic and immune reprogramming, unlike *Brca1*^−/−^ tumors
• An inflamed yet exhausted T cell microenvironment limits vaccine efficacy in *Brca1*^−/−^ tumors
• PARP inhibition and PD-1 blockade restore vaccine sensitivity in *Brca1*^−/−^ tumors

## Graphical abstract

**Figure:**
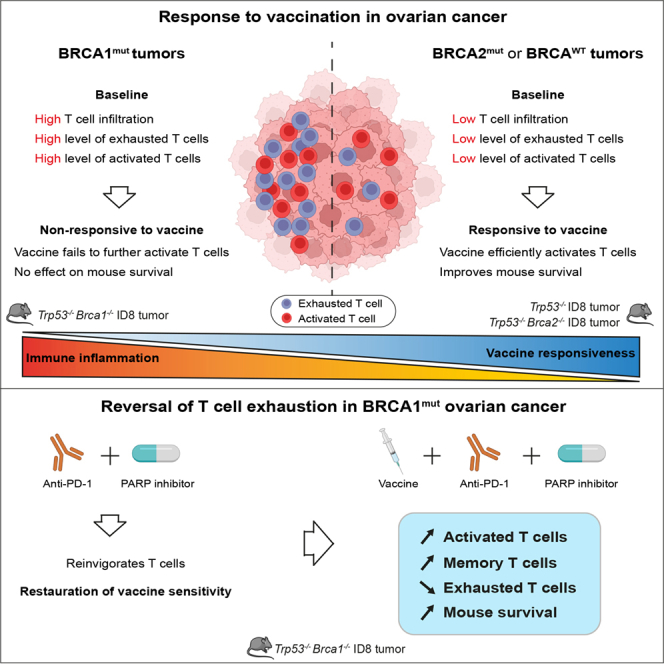

## Teaser

immunology; cell biology

## Introduction

Ovarian cancer (OC) is the deadliest gynecologic malignancy worldwide.^1^ The standard of care (SOC) treatments for OC combine debulking surgery with platinum-based chemotherapy.^2^^,^^3^ A significant advance in OC therapy has been the introduction of poly(ADP-ribose) polymerase inhibitors (PARPis) for patients with homologous recombination repair deficiency, particularly those carrying pathogenic *BRCA1/2* mutations.^4^ These mutations, which impair the error-free repair of double-strand DNA breaks by HR repair, are found in about 15%–25% of all OC patients.^5^ Despite initial responses to treatment often being favorable, high relapse rates and therapeutic resistance challenge the long-term survival of OC patients.^2^

Prior studies in OC patients have consistently reported a correlation between tumor-infiltrating lymphocytes (TILs), observed in most of the patients, and overall survival.^6^^,^^7^ Additionally, mutations in BRCA genes in OCs are associated with increased levels of TILs, immunoreactive gene signatures, and genomic instabilities.^8^^,^^9^ These observations support the use of immunotherapies to activate endogenous antitumor T cells in OC patients. Strategies include targeting immunomodulatory pathways with immune checkpoint inhibitors (ICIs), such as anti-programmed cell death 1 (PD-1) or its ligand (PD-L1).^10^^,^^11^ Another promising form of immunotherapy is vaccination, which can stimulate *de novo* T cell responses and enhance pre-existing ones.^10^ Recent research from our group has demonstrated that vaccinating OC patients with autologous dendritic cells (DCs) pulsed with oxidized autologous whole-tumor lysate (termed OCDC) successfully elicited T cell responses in these patients.^12^ Furthermore, combining OCDC vaccine with various agents that target the tumor microenvironment (TME), such as anti-vascular endothelial growth factor (VEGF)—a key immunosuppressive factor that promotes tumor angiogenesis—has resulted in improved survival outcomes for OC patients, as well as in murine models.^13^^,^^14^

While ICIs have been effective in treating melanoma or lung cancers, early results from immunotherapy in OC have shown only modest clinical benefit, with response rates around 10%–15% among unselected patients.^10^^,^^11^ This suggests the presence of significant immune resistance mechanisms. Although there is an increasing recognition of the role that BRCA mutations play in shaping tumor-immune interactions, their impact on vaccine efficacy remains largely unexplored. We hypothesized that the influence of BRCA mutations on T cell inflammation in OCs potentially contributes to cancer vaccine inefficacy. Therefore, we believe that understanding the mechanisms behind immunotherapy evasion in BRCA-mutated OCs could help identify which OC patients are most likely to respond to cancer vaccine.

In this study, we investigated how Brca mutations in murine OC cell lines affect their sensitivity to vaccination *in vivo*. We employed digital spatial profiling (DSP) to examine the transcriptomic profiles of tumor cell-enriched and immune cell-enriched compartments in our OC models. Unexpectedly, we found that loss of *Brca1*, but not *Brca2*, impairs the efficacy of cancer vaccination. This resistance is associated with a pre-existing immune-exhausted microenvironment. Importantly, we demonstrated that combining cancer vaccination with SOC or with ICI therapies restores vaccine sensitivity and reprograms the CD8 T cell response in *Brca1*-deficient tumors. Our findings underscore the importance of the OC genetic landscape in influencing immunotherapy response and support the development of rational combination strategies to overcome resistance and enhance vaccine efficacy in *BRCA1*-mutated OC.

## Results

### *Brca1* loss in murine ovarian tumors impairs vaccine effectiveness

**Figure 1:**
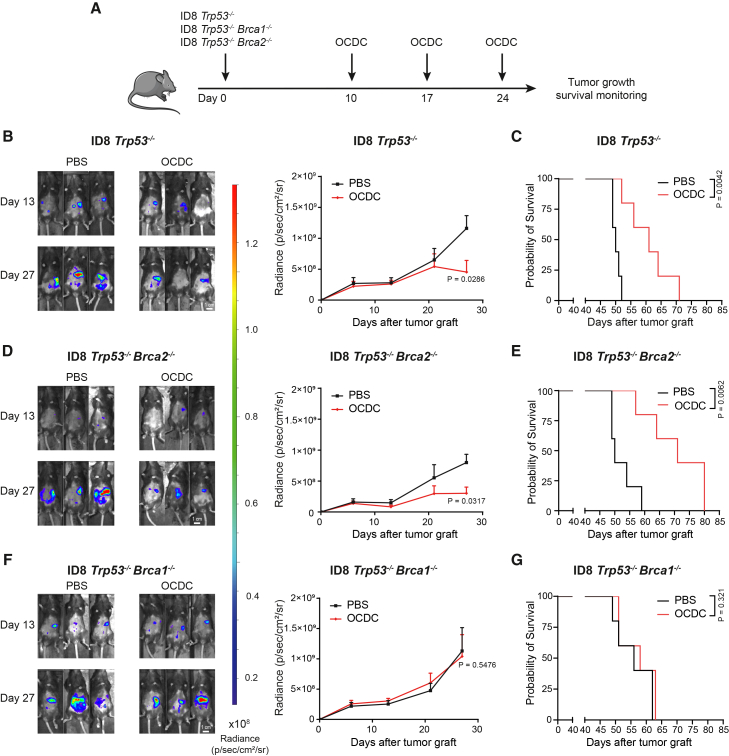
*Brca1* loss in ID8 tumors impairs vaccine effectiveness (A) Schematic representation of the OCDC vaccine schedule in C57BL/6J mice injected intraperitoneally with *Trp53*^−/−^, *Trp53*^−/−^*Brca1*^−/−^, or *Trp53*^−/−^*Brca2*^−/−^ ID8 tumors. (B, D, and F) Tumor growth kinetic of *Trp53*^−/−^ (B), *Trp53*^−/−^*Brca2*^−/−^ (D), and *Trp53*^−/−^*Brca1*^−/−^ (F) ID8 cancers. (Left) Representative bioluminescence (BLI) measurement and (right) kinetics of BLI measurements (*n* = 5 mice per group; graphs representative of three individual experiments; data are represented as mean ± SEM; unpaired nonparametric Mann-Whitney test). (C, E, and G) Comparison of survival curves of mice bearing *Trp53*^−/−^ (C), *Trp53*^−/−^*Brca2*^−/−^ (E) and *Trp53*^−/−^*Brca1*^−/−^ (G) ID8 tumors (*n* = 5 mice per group; graphs representative of three individual experiments; log-rank Mantel-Cox test).

To investigate the *in vivo* impact of Brca deletion on the sensitivity of OC to vaccine therapy, we adopted a syngeneic orthotopic ID8 mouse model deficient for *Trp53*, *Trp53* and *Brca1*, or *Trp53* and *Brca2*.^15^^,^^16^ Thus, C57BL/6J mice implanted with *Trp53*^−/−^, *Trp53*^−/−^*Brca1*^−/−^, or *Trp53*^−/−^*Brca2*^−/−^ ID8 cancer cells were vaccinated with OCDC (Figure 1A). OCDC vaccine therapy resulted in reduced tumor growth and improved mouse survival in both *Trp53*^−/−^ and *Trp53*^−/−^*Brca2*^−/−^ ID8 tumor models (Figures 1B–1E). Surprisingly, mice implanted with *Trp53*^−/−^*Brca1*^−/−^ ID8 tumors were resistant to the OCDC vaccine, both in terms of controlling tumor growth and mouse survival (Figures 1F and 1G). These data suggest that *Brca1* loss of function in ID8 ovarian tumors leads to vaccine inefficacy.

**Figure 2:**
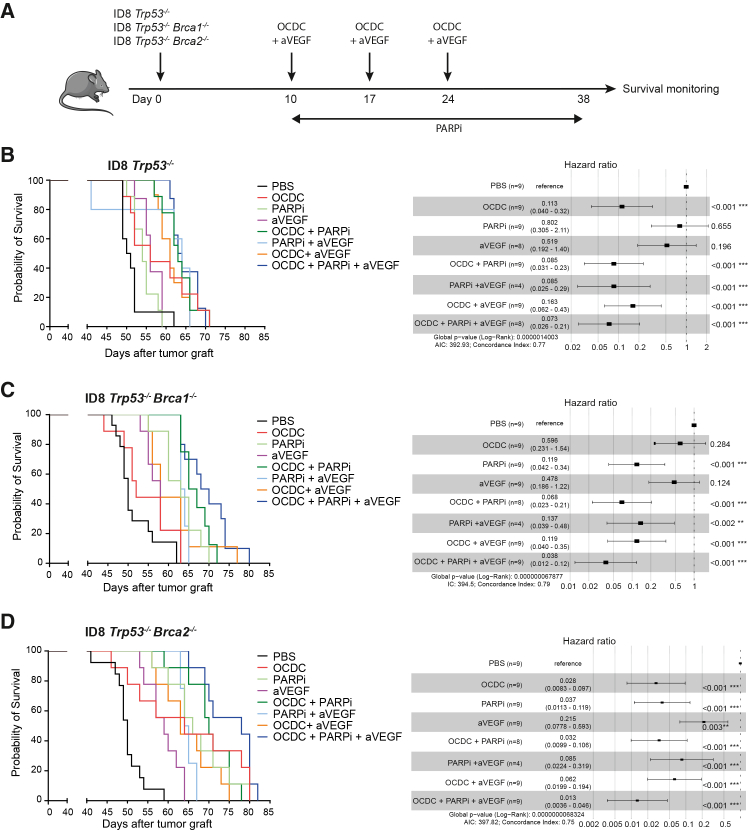
Combination with PARPi and anti-VEGF standards of care improves OCDC antitumor efficacy in the *Trp53*^−/−^*Brca1*^−/−^ ID8 tumor model (A) Schematic representation of the OCDC vaccine, PARPi and anti-VEGF treatments schedule in C57BL/6J mice injected intraperitoneally with *Trp53*^−/−^, *Trp53*^−/−^*Brca1*^−/−^, or *Trp53*^−/−^*Brca2*^−/−^ ID8 tumors. (B–D) (left) Comparison of survival curves and (right) forest plot for Cox proportional hazards model of mice bearing *Trp53*^−/−^ (B), *Trp53*^−/−^*Brca1*^−/−^ (C), and *Trp53*^−/−^*Brca2*^−/−^ (D) ID8 tumors (*n* = 4 to 9 mice per group; in forest plots squares indicate estimated hazard ratios, and horizontal lines represent 95% confidence intervals).

SOC for OCs with BRCA mutations currently involves targeted therapies, such as PARPi and anti-angiogenics.^2^^,^^17^ We thought to ask whether the combination of OCDC with such therapies would overcome vaccine resistance in the *Brca1*-deficient ovarian tumors. Mice orthotopically implanted with *Trp53*^−/−^, *Trp53*^−/−^*Brca1*^−/−^, or *Trp53*^−/−^*Brca2*^−/−^ ID8 cancer cells were treated with OCDC vaccine combined or not with an anti-VEGF and/or the PARPi olaparib (Figure 2A). As expected, PARPi single treatment improved mouse survival in the *Brca*-deficient ID8 models; however, anti-VEGF alone had only a weak effect on mouse survival in the *Trp53*^−/−^*Brca2*^−/−^ ID8 model. In the mice implanted with the OCDC-responding ID8 tumors (*Trp53*^−/−^ and *Trp53*^−/−^*Brca2*^−/−^), the benefit of the OCDC vaccine on survival was only enhanced in a proportion of mice by the combination with either PARPi or anti-VEGF, except with the triplet combination therapy (OCDC + PARPi + anti-VEGF) in the *Trp53*^−/−^*Brca2*^−/−^ ID8 model. Interestingly, in the OCDC-nonresponding ID8 model (*Trp53*^−/−^*Brca1*^−/−^), PARPi treatment slightly improved the survival of vaccinated mice. This beneficial effect was further increased in the mice treated with anti-VEGF, PARPi, and OCDC vaccine simultaneously (Figures 2B–2D). Taken together, our data demonstrated the inefficacy of cancer vaccine in *Brca1*-deficient ID8 ovarian tumors, which can be overcome by combining the vaccine with the SOC therapies PARPi and anti-VEGF.

### OCDC vaccine mediates biological reprogramming in *Trp53*^−/−^ and *Trp53*^−/−^*Brca2*^−/−^ ID8 tumor models

We sought to gain a deeper understanding of the transcriptomic profile of tumor cells, as well as the expression programs and states of TILs, in OCDC-nonresponding compared to OCDC-responding ID8 tumor models. Using the NanoString GeoMx platform, we performed DSP on the *Trp53*^−/−^, *Trp53*^−/−^*Brca1*^−/−^, or *Trp53*^−/−^*Brca2*^−/−^ ID8 tumor models upon vaccination combined with both PARPi and anti-VEGF. Tumor sections were stained with markers for T cells (CD3), for nuclei, for malignant cells (epithelial cell marker PanCK), and for monocytes/macrophages (CD68) (Figure S1A). We distinguished multiple regions of interest (ROIs) located inside or in the periphery of the tumors. In total, 648 ROIs were identified and selected based on immunostaining patterns for morphological markers and histology (Figure S1B). Next, each ROI was segmented into tumor cell-enriched (PanCK-high signal) or immune cell-enriched (PanCK-low signal) compartments (Methods and Figure S2).

**Figure 3:**
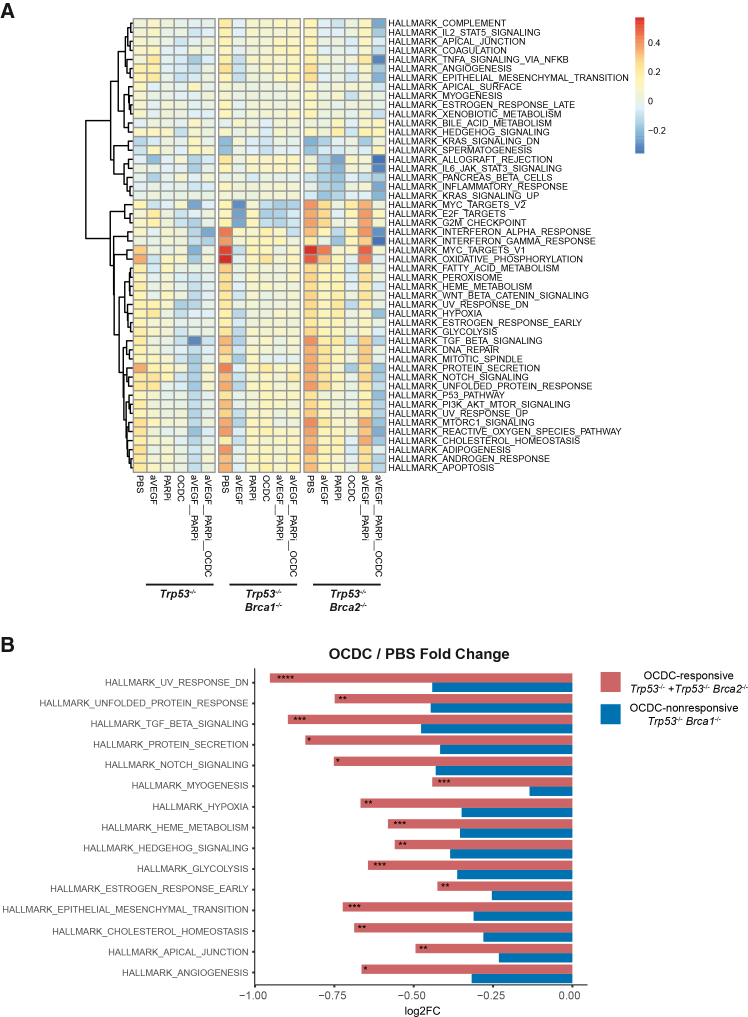
OCDC vaccine mediates biological reprogramming in *Trp53*^-/-^ and *Trp53*^-/-^*Brca2*^-/-^ ID8 tumor models (A) Heatmap of hallmark gene signatures of general biological processes in the tumor cell-enriched (panCK high) compartments of different treatment groups (PBS; anti-VEGF; PARPi; OCDC; anti-VEGF + PARPi; anti-VEGF + PARPi + OCDC), in the *Trp53*^−/−^, *Trp53*^−/−^*Brca1*^−/−^, and *Trp53*^−/−^*Brca2*^−/−^ ID8 tumor models. Scale bars represent median GSVA enrichment score. (B) Bar plots displaying the log2 fold change of general biological processes in the tumor cell-enriched compartments between OCDC and PBS groups, in the OCDC-responsive (*Trp53*^−/−^ and *Trp53*^−/−^*Brca2*^−/−^) and in the OCDC-nonresponsive (*Trp53*^−/−^*Brca1*^−/−^) ID8 tumor models (Wilcoxin test with Bonferroni correction). ∗*p* < 0.05, ∗∗*p* < 0.01, ∗∗∗*p* < 0.001, ∗∗∗∗*p* < 0.0001.

We performed gene set enrichment analysis (GSEA) using hallmark gene sets from the Molecular Signature Database. This analysis, conducted in both PanCK-low and PanCK-high compartments (Figure S3A), focused then on the tumor cell-enriched compartment (PanCK-high) to identify differences in gene expression between treatments in OCDC-responsive (*Trp53*^−/−^ and *Trp53*^−/−^*Brca2*^−/−^) and OCDC-nonresponsive (*Trp53*^−/−^*Brca1*^−/−^) ID8 tumors (Figure 3A). Our results revealed that the majority of the biologic programs were expressed at a higher level in PBS-treated samples of each ID8 tumor model and decreased after treatments, particularly after OCDC vaccine or the triplet therapy OCDC + anti-VEGF + PARPi. Interestingly, untreated *Trp53*^−/−^*Brca1*^−/−^ ID8 tumors showed a higher level of IFNα and IFNγ response pathways compared to *Trp53*^−/−^ and *Trp53*^−/−^*Brca2*^−/−^ ID8 tumors (Figure 3A). To better define the differences in gene expression, we compared hallmark gene sets in the OCDC vaccine and PBS groups that were significantly modulated upon vaccination in OCDC-responsive tumors or in OCDC-nonresponsive tumors (Figure 3B). We observed a significant negative enrichment of several gene sets, including pathways involved in TGF-beta (TGFβ) signaling, glycolysis, cholesterol homeostasis, epithelial to mesenchymal transition (EMT), unfolded protein response (UPR), or protein secretion pathways, only in OCDC-responsive tumors, suggesting a disruption of tumor metabolism upon immune attack, leading to a reduction of proliferation and invasiveness (EMT) or immune evasion (TGFβ). In contrast, OCDC-nonresponsive tumors maintained their metabolic and proliferative capacity. Similar patterns were observed at the gene-level when the expression of representative genes composing these key pathways were analyzed (Figure S3B).

### Predominance of activated and exhausted T cell gene signatures in mice implanted with *Trp53*^−/−^*Brca1*^−/−^ ID8 tumor model

**Figure 4:**
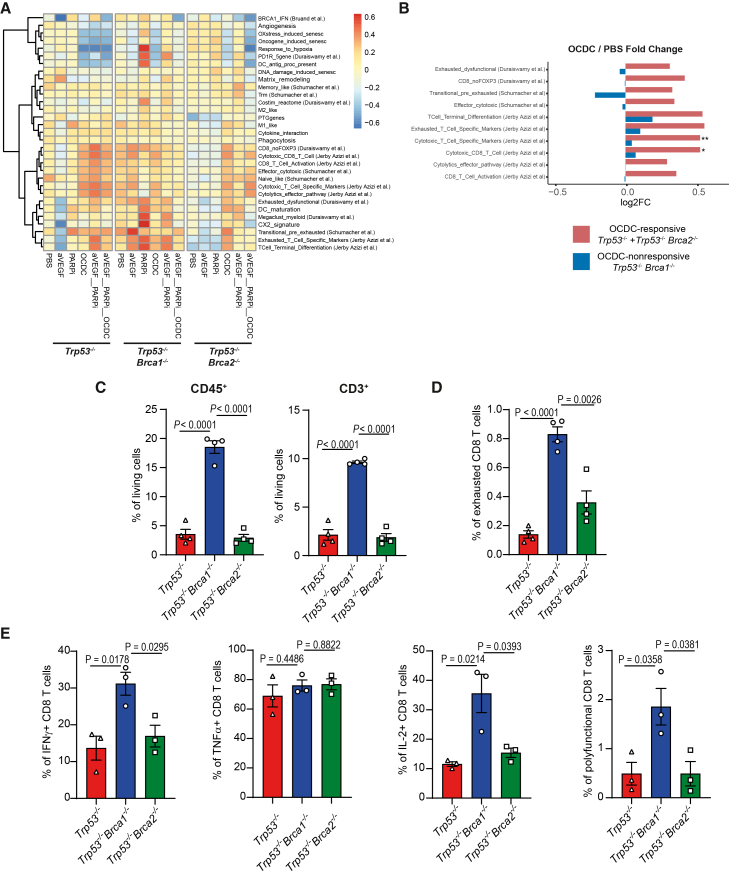
Predominance of activated and exhausted T cells in mice implanted with *Trp53*^−/−^*Brca1*^−/−^ ID8 tumor model (A) Heatmap of immune and T cell subsets gene signatures in the immune cell-enriched (panCK low) compartments of different treatment groups (PBS; anti-VEGF; PARPi; OCDC; anti-VEGF + PARPi; anti-VEGF + PARPi + OCDC), in the *Trp53*^−/−^, *Trp53*^−/−^*Brca1*^−/−^, and *Trp53*^−/−^*Brca2*^−/−^ ID8 tumor models. Scale bars represent median GSVA enrichment score. (B) Bar plots displaying the log2 fold change of T cell subsets gene signatures in the immune cell-enriched compartments between OCDC and PBS groups, in the OCDC-responsive (*Trp53*^−/−^ and *Trp53*^−/−^*Brca2*^−/−^) and in the OCDC-nonresponsive (*Trp53*^−/−^*Brca1*^−/−^) ID8 tumor models (Wilcoxin test with Bonferroni correction). ∗*p* < 0.05, ∗∗*p* < 0.01. Quantification of (C) CD45^+^ immune cells (left) and CD3^+^ T cells (right) in tumors, and of (D) PD-1^hi^Tim-3^+^Lag-3^+^TIGIT^+^CD39^+^Eomes^+^TOX^hi^ exhausted CD8 T cells and (E) IFNγ-, TNFα-, or IL-2-producing CD8 T cells and polyfunctional CD8 T cells infiltrated in *Trp53*^−/−^, *Trp53*^−/−^*Brca1*^−/−^, and *Trp53*^−/−^*Brca2*^−/−^ ID8 ascites by flow cytometry (*n* = 3 to 4; graphs representative of two individual experiments; data are represented as mean ± SEM; unpaired parametric *t* test).

Next, we aimed to determine whether *Brca* deletion would impact T cell activation and responses to cancer vaccine. To study the impact of OCDC vaccine, PARPi, and anti-VEGF on immune cell gene signatures in OCDC-responsive and OCDC-nonresponsive ID8 tumors, we utilized the DSP in both PanCK-low and PanCK-high compartments and performed unsupervised clustering based on an in-house collection of published gene signatures that capture more in-depth immune responses and T cell activation (Methods, Table S1 and Figure S4A).^18^^,^^19^^,^^20^^,^^21^^,^^22^ Most notably, we observed that the immune cell-enriched compartments (PanCK-low) in PBS-treated *Trp53*^−/−^*Brca1*^−/−^ tumors already displayed high levels of activated and exhausted T cell signatures compared to *Trp53*^−/−^ or *Trp53*^−/−^*Brca2*^−/−^ ID8 tumors (Figures 4A and S4B). After the OCDC vaccine, a similar activated and exhausted T cell status was observed in all ID8 tumor models (Figures 4A and S4B). Consequently, the fold changes in T cell activation and exhaustion signatures comparing the OCDC vaccine and PBS groups were superior in the OCDC-responsive *Trp53*^−/−^ and *Trp53*^−/−^*Brca2*^−/−^ compared to the OCDC-nonresponsive *Trp53*^−/−^*Brca1*^−/−^ ID8 tumor models (Figures 4B and S5). Importantly, these results were validated by flow cytometry. First, we observed at baseline higher CD45^+^ immune cell and CD3^+^ T cell infiltration in mice implanted with *Trp53*^−/−^*Brca1*^−/−^ tumors compared to those with *Trp53*^−/−^ or *Trp53*^−/−^*Brca2*^−/−^ ID8 tumors, suggesting a more inflamed TME in *Brca1*-deficient tumors (Figure 4C). The CD8 T cells from *Trp53*^−/−^*Brca1*^−/−^ tumors exhibited significantly higher co-expression of multiple exhaustion-associated markers, PD-1^hi^Tim-3^+^Lag-3^+^TIGIT^+^CD39^+^Eomes^+^TOX^hi^, compared to Trp53^−/−^ and Trp53^−/−^Brca2^−/−^ tumors (Figure 4D). In parallel, we assessed T cell functional status by measuring IFNγ, TNFα, and IL-2 cytokine production. We found that *Trp53*^−/−^*Brca1*^−/−^ tumors exhibited a higher level of CD8 T cells producing IFNγ and IL-2, and a higher level of polyfunctional CD8 T cells (producing simultaneously IFNγ, TNFα, and IL-2) compared to *Trp53*^−/−^ and *Trp53*^−/−^*Brca2*^−/−^ models (Figure 4E). Similar observations were made in the spleens of ID8-bearing mice (Figure S6). These results suggest the presence of an immunologically active and chronic immune-inflammatory environment in the tumor of the vaccine-nonresponsive *Trp53*^−/−^*Brca1*^−/−^ ovarian ID8 tumor model.

### Anti-PD-1 combined with PARPi restores the anti-tumor efficacy of cancer vaccine and overcomes immune exhaustion in the *Brca1*-deficient ID8 tumor model

**Figure 5:**
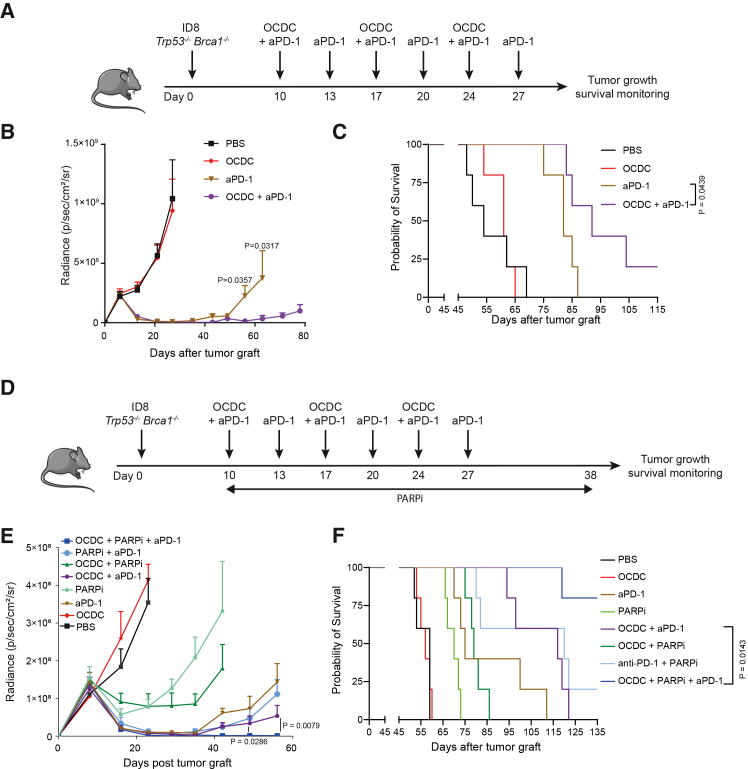
Anti-PD-1, PARPi and OCDC vaccine triplet therapy yields durable tumor control in the *Brca1*-deficient ID8 tumor model (A) Schematic representation of the OCDC vaccine and anti-PD-1 treatments schedule in C57BL/6J mice injected intraperitoneally with *Trp53*^−/−^*Brca1*^−/−^ ID8 tumors. (B) Tumor growth kinetics by bioluminescence measurements (*n* = 8 mice per group; data are represented as mean ± SEM; unpaired nonparametric Mann-Whitney test). (C) Comparison of survival curves (*n* = 5 mice per group; graph representative of three individual experiments; log-rank Mantel-Cox test). (D) Schematic representation of the OCDC vaccine, PARPi and anti-PD-1 treatments schedule in C57BL/6J mice injected intraperitoneally with *Trp53*^−/−^*Brca1*^−/−^ ID8 tumors. (E) Tumor growth kinetics by bioluminescence measurements (*n* = 5 to 8 mice per group; data are represented as mean ± SEM; unpaired nonparametric Mann-Whitney test). (F) Comparison of survival curves (*n* = 5 mice per group; graph representative of three individual experiments; log-rank Mantel-Cox test).

The pre-existing immune-exhausted environment observed in the *Trp53*^−/−^*Brca1*^−/−^ ID8 tumor model suggests that combining immune checkpoint blockade therapy with a cancer vaccine would prevent vaccine evasion of *Brca1*-mutated ovarian tumors. Thus, C57BL/6J mice implanted with *Trp53*^−/−^*Brca1*^−/−^ ID8 cancer cells were vaccinated with OCDC in combination with anti-PD-1 therapy (Figure 5A). Importantly, we demonstrated that the OCDC vaccine, which was ineffective in the *Trp53*^−/−^*Brca1*^−/−^ ID8 tumor model, significantly decreased the tumor growth rate when combined with anti-PD-1 (Figure 5B). Furthermore, the combination led to improved mouse survival (Figure 5C). Notably, compared to OCDC-responding *Trp53*^−/−^ and *Trp53*^−/−^*Brca2*^−/−^ ID8 models, we observed that anti-PD-1 monotherapy was more effective in controlling tumor growth in the *Trp53*^−/−^*Brca1*^−/−^ ID8 model (Figures 5B and S7A). Although PD-L1 expression was observed across all tumor models, its expression was higher in *Trp53*^−/−^*Brca1*^−/−^ ID8 cells compared to *Trp53*^−/−^ and *Trp53*^−/−^*Brca2*^−/−^ ID8 cells (Figure S8S7B). Nonetheless, the combination of OCDC vaccine with anti-PD-1 significantly decreased the tumor growth rates in all models (Figures 5B and S7A).

**Figure 6:**
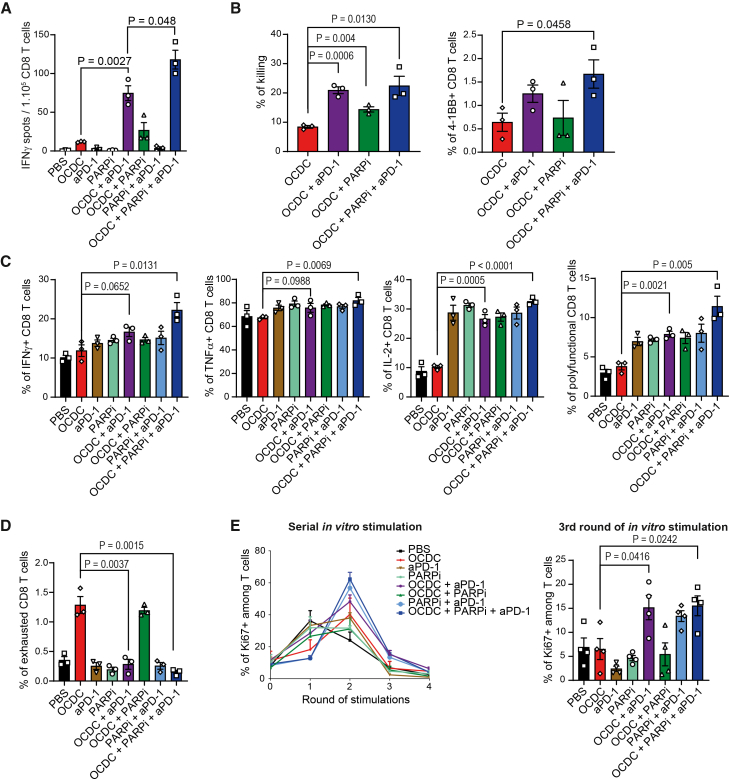
Anti-PD-1, PARPi, and OCDC vaccine triplet therapy overcomes CD8 T cell exhaustion in *Brca1*-deficient ID8 tumor model C57BL/6J mice injected intraperitoneally with *Trp53*^−/−^*Brca1*^−/−^ ID8 tumors were treated with OCDC vaccine, PARPi and anti-PD-1. (A) Functional analysis of ID8-specific CD8 T cells measured *ex vivo* in the splenocytes by IFNγ-ELISpot (*n* = 3; graph representative of three individual experiments; data are represented as mean ± SEM; unpaired parametric *t* test). (B) T cells from spleen of vaccinated mice were co-cultured with *Trp53*^−/−^*Brca1*^−/−^ ID8 tumor cells (ratio 2 T cells:1 tumor cell) *in vitro*. (Left) Killing measurements after 16 h co-cultures. (Right) Quantification of 4-1BB^+^ CD8 T cells by flow cytometry (*n* = 3; graph representative of two individual experiments; data are represented as mean ± SEM; unpaired parametric *t* test). (C) Quantification of IFNγ-, TNFα-, or IL-2-producing CD8 T cells and of polyfunctional CD8 T cells by flow cytometry in the splenocytes. (D) Quantification of PD-1^hi^Tim-3^+^Lag-3^+^TIGIT^+^CD39^+^Eomes^+^TOX^hi^ exhausted CD8 T cells by flow cytometry in the splenocytes. (E) T cells from mice treated with OCDC, PARPi or anti-PD-1 were activated *in vitro* with anti-CD3/CD28 activating antibodies. (Left) Longitudinal quantification of Ki67^+^ CD8 T cells by flow cytometry and (right) quantification of Ki67^+^ CD8 T cells by flow cytometry after three rounds of stimulation (*n* = 3; graph representative of two individual experiments; data are represented as mean ± SEM; unpaired parametric *t* test).

Interestingly, we showed that PD-L1 expression was increased upon PARPi treatment in *Trp53*^−/−^*Brca1*^−/−^ ID8 (Figure S8). Thus, we asked whether combining the OCDC vaccine with PARPi and anti-PD-1 would further improve the anti-tumoral efficacy observed with the OCDC vaccine and anti-PD-1 in the *Trp53*^−/−^*Brca1*^−/−^ ID8 tumor model (Figure 5D). No significant body weight loss was observed following treatment, suggesting a safety profile of the triplet therapy (Figure S9). We demonstrated that combining PARPi with anti-PD-1 enhanced tumor growth control and improved mouse survival compared to each treatment alone (Figures 5E, 5F, and S10). Strikingly, the addition of PARPi to the OCDC vaccine and anti-PD-1 significantly abrogated tumor growth, resulting in the survival of 80% of the mice (Figures 5E and 5F). Notably, the anti-tumoral benefit of combining PARPi and anti-PD-1 to OCDC was correlated with an increase in ID8-specific IFNγ-producing CD8 T cells (Figure 6A), which displayed an increased *in vitro* tumor killing capability and activation profile when co-cultured with *Trp53*^−/−^*Brca1*^−/−^ ID8 tumors (Figure 6B). Furthermore, the CD8 T cells from the triplet therapy group showed a higher level of polyfunctionality, with the ability to produce simultaneously IFNγ, TNFα and IL-2 (Figure 6C). Interestingly, we observed a significant increase in central memory CD8 (TCM) population in mice treated with triplet therapy or with OCDC + anti-PD-1 compared to control treatment groups (Figure S11), suggesting that the therapeutic benefit was associated with the generation of a memory CD8 T cell pool with potential long-term protective capacity. Importantly, the enhanced effector CD8 T cell responses in the triplet therapy were associated with a reduced frequency of PD-1^hi^Tim-3^+^Lag-3^+^TIGIT^+^CD39^+^Eomes^+^TOX^hi^ exhausted CD8 T cells, compared to the elevated exhaustion phenotype induced by OCDC vaccination alone (Figure 6D). To further characterize the CD8 T cell exhaustion at a functional level, we performed serial *in vitro* restimulation assays. T cells isolated from treated mice were subjected to repeated stimulation with anti-CD3/CD28, and proliferation was assessed by Ki67 expression. We found that T cells from mice treated with anti-PD-1 and PARP inhibitor exhibited enhanced proliferative capacity upon several rounds of restimulation compared to T cells from OCDC-treated mice, indicating reduced exhaustion (Figure 6E).

In summary, we showed that *Brca1*-deficient ovarian murine tumors are resistant to cancer vaccine, but by overcoming the immune-inflamed and exhausted TME with immune checkpoint blockade therapy, the efficacy of cancer vaccine can be restored.

## Discussion

TILs have been associated with a better outcome in OC, suggesting that cancer immunotherapy might improve survival.^6^^,^^7^ However, the success of ICIs and cancer vaccines has been generally limited in OC patients.^10^^,^^11^ Efforts should thus focus on defining biomarkers that could help identify which OC patients are most likely to respond to immunotherapies. In this study, we investigated how the genomic landscape of OC differentially affects response to cancer vaccine. Surprisingly, we found that contrary to the loss of *Brca2*, the loss of *Brca1* in ovarian tumors induces a striking resistance to cancer vaccine. This was observed after vaccination of mice implanted with *Trp53*^−/−^, *Trp53*^−/−^*Brca1*^−/−^, or *Trp53*^−/−^*Brca2*^−/−^ ID8 ovarian murine tumor model. Following a DC-based vaccine, a decreased tumor growth rate and improved mouse survival were observed in mice implanted with *Trp53*^−/−^ or *Trp53*^−/−^*Brca2*^−/−^ ID8 tumors; however, no benefit was observed in *Trp53*^−/−^*Brca1*^−/−^ ID8-bearing mice. Similar results have been reported *in vivo* in a murine breast cancer model, where mutations in *Brca2* were associated with a superior response to anti-PD-1 and anti-CTLA-4 compared to those in *Brca1*.^23^ More recently, Park et al. described *in vitro* that CD8 TILs from wild-type *BRCA1/2* epithelial OC patients were more sensitive to a reinvigoration mediated by anti-PD-1 compared to CD8 TILs from mutated *BRCA1/2*.^24^

To investigate the mechanisms implicated in OC sensitivity to vaccine, we performed DSP to study the tumor cell and immune cell-enriched compartments in the *Trp53*^−/−^, *Trp53*^−/−^*Brca1*^−/−^, and *Trp53*^−/−^*Brca2*^−/−^ ID8 tumor models. Importantly, we observed that vaccine efficacy in the *Trp53*^−/−^ and *Trp53*^−/−^*Brca2*^−/−^ tumor models is associated with a biological reprogramming of the tumor. Upon vaccination in OCDC-responsive tumors, a suppressed cholesterol homeostasis and glycolysis demonstrated a shift in metabolism, suggesting a higher sensitivity to therapy.^25^ A decline in TGFβ signaling and EMT pathway activity indicates a lower immunosuppressive environment and reduced tumor invasion potential.^26^ The downregulation of UPR and protein secretion pathways highlights a diminution in endoplasmic reticulum stress, promoting tumor immunogenicity.^27^ In contrast, the OCDC-nonresponsive *Trp53*^−/−^*Brca1*^−/−^ ID8 tumor model failed to exhibit these pathway shifts upon vaccination, suggesting that tumors deficient in *Brca1* can maintain an immunosuppressive, metabolically robust, and invasive phenotype, thereby leading to immune evasion mechanisms.

Furthermore, our results confirmed the higher infiltration of T cells observed in tumors deficient in *Brca1* compared to those lacking in *Brca2* or with a wild-type *Brca*.^28^ Previous work from our group has shown that BRCA1-mutated high-grade serous ovarian cancers (HGSOCs) are associated with a higher frequency of CD8 T cell tumor infiltration and IFN signature compared to homologous recombination-proficient patients.^18^ Similar observations were made by Alvaro et al. that performed sequencing analysis of 2745 HGSOC tumor samples and demonstrated higher T cell inflamed and IFNγ scores, and higher PD-L1 expression in BRCA-mutated tumors.^28^ Consistent with this, our group more recently analyzed a large-scale cohort of 697 human ovarian cancer samples and showed that tumors can be subdivided in four immune phenotypes based on TIL infiltration (pure-inflamed, mixed-inflamed, excluded or desert) and that this categorization was linked to prognosis and treatment response. Interestingly, BRCA-mutated tumors are mostly (67%) composed of inflamed (purely and mixed-inflamed) immune phenotype.^29^ Despite this increased T cell infiltration, responses to immunotherapies in ovarian or breast cancer patients with *BRCA1* germline mutations have remained low,^23^^,^^30^ suggesting that *BRCA1* deficiency may drive immunoregulatory processes that limit responses to these therapies. Concordant with these studies exploring *BRCA1*-mutated tumors, our analyses indicate that tumor-response to IFNα and IFNγ signature pathways, and gene expression programs related to activated or exhausted T cell populations were enriched specifically in *Trp53*^−/−^*Brca1*^−/−^ tumors relative to *Trp53*^−/−^ or *Trp53*^−/−^*Brca2*^−/−^ ID8 tumors. We demonstrated that post-vaccination, the activation and exhaustion T cell programs were increased in the OCDC-responsive tumors to reach a similar level as the *Trp53*^−/−^*Brca1*^−/−^ OCDC-nonresponsive tumors. While pre-existing TILs are crucial for the efficacy of immunotherapies,^31^ our work suggests that, paradoxically, upon vaccination, the function of highly pre-activated TILs cannot be further enhanced. We suggest that an overly activated yet exhausted T cell milieu can hinder the success of cancer vaccines in BRCA1-deficient ovarian tumors, necessitating combination therapies to improve vaccine efficacy.

While cancer vaccination is a potential strategy to promote T cell infiltration in immune-excluded or immune-desert (“cold”) tumors, immune-inflamed (“hot”) tumors—characterized, among other things, by increased numbers of PD-1^+^ and GZMB^+^ T cells—are considered a prerequisite for response to ICIs.^11^ Here, in an attempt to overcome the resistance to cancer vaccine caused by pre-existing exhausted T cells in the *Trp53*^−/−^*Brca1*^−/−^ ID8 tumor model, we combined the OCDC vaccine with anti-PD-1. A particularly important finding was that anti-PD-1, in addition to being effective alone, could restore vaccine responsiveness in the *Brca1*-deficient ID8 model. This combination not only slowed tumor growth and improved survival but also reprogrammed the T cell compartment, increasing tumor-specific IFNγ-producing T cells while reducing exhaustion markers. Combining ICIs with cancer vaccines in ovarian settings has shown promising results in preclinical studies.^32^^,^^33^^,^^34^^,^^35^ Multiple clinical trials are also investigating this combination in OC patients.^36^^,^^37^^,^^38^ These trials show promising safety and efficacy results; however, the genetic landscape of OC is not considered. We suggest that *BRCA1* and *BRCA2* mutations may not be functionally equivalent in shaping the tumor immune environment and response to immunotherapy.

Prior work from our group has dissected the DNA-damage response pathway and has shown in *BRCA1*-deficient ovarian cancer that cell-autonomous activation of DNA-sensing cyclic guanosine monophosphate-AMP synthase (cGAS) and stimulator of interferon genes (STING) and chronic type I IFN signaling can drive both tumor-intrinsic immunoreactivity and immune resistance, including upregulation of PD-L1 and other checkpoint molecules.^18^ These observations supported a model in which *BRCA1* loss promotes a persistent STING-IFN axis that maintains T cell infiltration but favors terminal or near-terminal exhaustion, whereas *BRCA2* loss, despite also increasing genomic instability, may engage these pathways less strongly or with different kinetics. This work suggested that VEGF blockade could phenocopy STING genetic loss and could synergize with PARPi and ICIs to control the growth of *Trp53*^−/−^*Brca1*^−/−^ ID8 tumors. We showed here that the addition of PARPi and anti-VEGF restored vaccine efficacy in *Brca1*-deficient ID8 tumors, highlighting the potential for integrating immunotherapy with established SOC treatments in *BRCA1*-mutated OC.

Confirming previous observations in breast cancers,^39^ we have demonstrated here that PARPi can upregulate PD-L1 expression on *Brca1*-deficient ovarian tumor cells, thereby attenuating antitumor immunity. We showed that combining anti-PD-1 and PARPi improves tumor control in the *Trp53*^−/−^*Brca1*^−/−^ ID8 tumor model. Most notably, we demonstrated that the triplet combination of OCDC vaccine, anti-PD-1, and PARPi resulted in near-complete tumor eradication and long-term survival in most treated mice. Our data suggest that BRCA status should be considered not only in the context of DNA repair-targeted therapies but also when designing immunotherapy regimens. Thus, targeting both DNA damage response pathways and immune exhaustion can synergize to unlock the full potential of cancer vaccines in resistant *BRCA1*^−/−^ tumors. We recommend that *BRCA1* genetic deficiency, baseline high immune activation/exhaustion states, and interferon and PD-L1 signaling could serve as candidate biomarkers to stratify ovarian cancer patients and guide treatment. Our proposed strategy could be integrated into current clinical paradigms, which include cytoreductive surgery and platinum-based chemotherapy, followed by PARPi maintenance therapy in BRCA-mutated or homologous recombination-deficient disease.^2^^,^^3^ Building on this framework, we propose a model in which vaccination is administered in combination with PARP inhibition and PD-1 blockade in the maintenance setting, where tumor burden is low and immune-based strategies may be more effective. In the same line, Higuchi et al. demonstrated the synergistic efficacy of combining an ICI agent with PARPi, while Bruand et al. showed that adding anti-VEGF to this combination further enhanced the antitumor efficacy in a *Brca1*^−/−^ murine ovarian model.^18^^,^^40^ In BRCA wild-type OC patients,^41^ or in unselected BRCA status OC patients,^42^ clinical studies are evaluating the potential of this triplet maintenance therapy, comprising PARPi, ICI, and anti-VEGF, suggesting the feasibility of such multi-agent approaches. In summary, we demonstrated here that *Brca1*-deficient ovarian tumors exhibit intrinsic resistance to cancer vaccines due to a pre-existing activated yet exhausted immune microenvironment. This resistance can be overcome through rational combination therapies that include immune checkpoint blockade or PARP inhibition, paving the way for more effective immunotherapeutic approaches in this challenging subset of OC.

### Limitations of the study

Our findings are based on syngeneic ovarian murine ID8 models, which do not fully recapitulate the complexity and heterogeneity of human ovarian cancer. Although we and others have previously reported an increased inflamed immune phenotype in *BRCA1*-mutated ovarian cancer patients,^18^^,^^28^^,^^29^ we acknowledge that direct validation in human datasets or human-derived systems such as patient-derived xenograft or organoids, particularly using samples from patients treated with immunotherapies, would further strengthen the translational relevance of our study. Furthermore, safety remains a key consideration for the clinical implementation of multi-agent combination therapies, and formal toxicological evaluation will be required to assess tolerability in clinical settings.

## Resource availability

### Lead contact

Further information and requests for resources and reagents should be directed to and will be fulfilled by the lead contact, Lana Kandalaft (lkandalaft@swissmedical.net).

### Materials availability

This study did not generate new unique reagents.

### Data and code availability

• The data supporting the findings of this study have been deposited in the Gene Expression Omnibus (GEO) and are publicly available. The GEO accession number is listed in the key resources table.
• This study does not report original code.
• Any additional information required to reanalyze the data reported in this study is available from the lead contact upon request.

## Acknowledgments

We thank Anne-Laure Huguenin, Mathieu Desbuisson and Marina Alexandre Gaveta for technical assistance. This work was supported by the Ludwig Institute for Cancer Research (LICR). This study benefited from the excellent technical support of the AIVC, IVIF, and FCF facilities in Agora Translational Cancer Research Center.

## Author contributions

L.E.K. conceived, designed, and supervised the project. L.B., C.L.-L.C., R.M.S., M.M.C., and R.R. performed the experiments and analyzed the results. J.T. and S.T. performed the digital spatial profiling experiments, A.M. analyzed the digital spatial profiling experiments. L.B. wrote the original draft of the manuscript. L.B., A.M., D.D.-L., and L.E.K. reviewed and edited the manuscript. L.E.K. acquired the fundings. All authors read and approved the final version of the manuscript.

## Declaration of interests

The authors declare no competing interests.

## STAR★Methods

### Key resources table

**Table:** 

REAGENT or RESOURCE	SOURCE	IDENTIFIER
**Antibodies**		

InVivoMAb anti-mouse PD-1 (clone: RMP1-14)	BioXcell	Cat # BE0146, RRID: AB_10949053
Anti-VEGF (clone B20–4.1.1)	Genentech	A kind gift from Genentech
Purified anti-mouse CD16/32 Antibody	Biolegend	Cat # 101301, RRID: AB_312800
Pacific blue anti-CD4 (clone RM4-5)	Biolegend	Cat # 100531, RRID: AB_493374
BUV395 anti-CD8 (clone 53–6.7)	BD Biosciences	Cat # 563786, RRID: AB_2732919
APC anti-LAG-3 (clone C9B7W)	Biolegend	Cat # 125210, RRID: AB_10639727
RB780 anti-PD-1 (clone J43)	BD Biosciences	Cat # 755328, RRID: AB_3687733
BV605 anti-Tim-3 (RMT3-23)	Biolegend	Cat # 119721, RRID: AB_2616907
Pecy7 anti-CD39 (clone Duha59)	Biolegend	Cat # 143806, RRID: AB_2563394
PE anti-TIGIT (clone 1G9)	Biolegend	Cat # 142104, RRID: AB_10933258
AF488 anti-TOX (clone NAN448B)	BD Biosciences	Cat # 569821, RRID: AB_3685349
BUV737 anti-EOMES (clone X4-83)	BD Biosciences	Cat # 567170, RRID: AB_2916487
BUV395 anti-CD4 (clone RM4-5)	BD Biosciences	Cat # 740208, RRID: AB_2734761
FITC anti-CD8 (clone KT15)	ThermoFisher Scientific	Cat # MA516759, RRID: AB_2538242
Brilliant Violet 421 anti-IL-2 (clone JES6-5H4)	Biolegend	Cat # 503826, RRID: AB_2650897
PE anti-TNFα (clone MP6-XT22)	ThermoFisher Scientific	Cat # 12-7321-82, RRID: AB_466199
APC anti-CD8 (clone 53–6.7)	Biolegend	Cat # 100712, RRID: AB_312751
Brilliant Violet 711 anti-IFNγ (clone XMG1.2)	Biolegend	Cat # 505836, RRID: AB_2650928
Brilliant Violet 421 anti-PD-L1 (clone 10F.9G2)	Biolegend	Cat # 124315, RRID: AB_10897097
APC anti-CD4 (clone GK1.5)	Biolegend	Cat # 100412, RRID: AB_312697
Brilliant Violet 711 anti-CD62L (clone MEL-14)	Biolegend	Cat # 104445, RRID: AB_2564215
Brilliant Violet 421 anti-CD44 (clone IM7)	Biolegend	Cat # 103040, RRID: AB_2616903
PE anti-41-BB (clone 17B5)	Biolegend	Cat # 106105, RRID: AB_2205693
APC anti-Ki67 (clone 16A8)	Biolegend	Cat # 652405, RRID: AB_2561929
BUV737 anti-CD3 (clone 145-2C11)	BD Biosciences	Cat # 612771, RRID: AB_2870100
CD40 Monoclonal Antibody (HM40-3)	ThermoFisher Scientific	Cat # 14-0402-86, RRID: AB_467230
APC anti-CD45 (clone: 30-F11)	Biolegend	Cat # 103112, RRID: AB_312977
Purified anti-mouse CD3e (clone 145-2C11)	BD Biosciences	Cat # 567115, RRID: AB_2916449
Purified anti-mouse CD28 (clone D665)	BD Biosciences	Cat # 566883, RRID: AB_2869934
AF488 anti-CD3 (clone CD3-12)	Bio-rad	Cat # MCA1477A488, RRID: AB_10844215
AF594 anti-CD68 (clone KP1)	Santa Cruz	Cat # sc-, 20060AF594, RRID: AB_3751055
AF647 anti-cytokeratin, pan (clone AE-1/AE-3)	Novus	Cat # NBP2-33200AF647, RRID: AB_3284602

**Chemicals, peptides, and recombinant proteins**		

DNAse I	Roche	Cat # 10104159001
Liberase™ TM Research Grade	Roche	Cat # 5401119001
Liberase™ TH Research Grade	Roche	Cat # 5401135001
Olaparib	APExBIO	Cat # A4154
Recombinant Mouse IFNγ	Peprotech	Cat # 315-05
CpG ODN 1668	InvivoGen	Cat # tlrl-1668
PMA/Ionomycin	eBioscience	Cat # 00-4970-93
AnnexinV FITC conjugate	Biolegend	Cat # 640906
DAPI	Sigma	Cat #D9542-5 MG
Syto™83 Orange fluorescent nucleic acid stain	ThermoFischer Scientific	Cat #S11364
D-luciferin	Biosynth	Cat # L-8220
Hygromycin	Millipore	Cat # 400052
DMEM	ThermoFischer Scientific	Cat # 41966-029
RPMI 1640	ThermoFischer Scientific	Cat # 1870010
Poly(ethylene glycol) 300	Sigma-Aldrich	Cat # 202371
IMDM	ThermoFischer Scientific	Cat # 12440061
Mouse GM-CSF Recombinant Protein	Peprotech	Cat # 315-03
Mouse IL-4 Recombinant Protein	Peprotech	Cat # 214-14
Cell Activation Cocktail (with Brefeldin A)	Biolegend	Cat #423304
Brefeldin A Solution (1,000X)	Biolegend	Cat # 420601
LPS-EB	Invivogen	Cat # tlrl-eblps

**Critical commercial assays**		

Easysep mouse T cells	Stemcell	Cat #19851
Easysep mouse CD8^+^ T cell isolation kit	Stemcell	Cat #19853
Easysep mouse B cell isolation kit	Stemcell	Cat #19854
Zombie UV™ Fixable Viability Kit	Biolegend	Cat #423107
Fixation/Permeabilization Kit	BD Biosciences	Cat # 554714
IFNγ-ELISpot	Diaclone	Cat # 862.031.010P
GeoMx mouse whole transcriptome atlas	Bruker	Cat # 121401103
GeoMx RNA slide prep kit	Bruker	Cat # 121300313
GeoMx DSP instrument buffer kit	Bruker	Cat # 100474
GeoMx Seq code pack: A, B, C & D	Bruker	Cat # 121400205

**Deposited data**		

Analyzed DSP data	This paper	GEO: GSE309310

**Experimental models: Cell lines**		

ID8 *Trp53*^−/−^, ID8 *Trp53*^−/−^*Brca1*^−/−^ and ID8 *Trp53*^−/−^*Brca2*^−/−^	Prof. Iain A. McNeish lab	(Walton et al.^15^; Walton et al.^16^); https://doi.org/10.1158/0008-5472.CAN-16-1272; https://doi.org/10.1038/s41598-017-17119-1

**Experimental models: Organisms/strains**		

Mouse: C57BL/6J wt	Envigo	057

**Software and algorithms**		

Graphpad Prism (v.9.4.0)	Dotmatics	https://www.graphpad.com/features
FlowJo (version 10.7.2)	BD	https://www.flowjo.com
R (version 4.0)	The R Foundation	https://www.r-project.org/
bcl2fastq program	Illumina	https://www.illumina.com
Nanostring’s GeoMx DnD pipeline (v2.0.0.16)	Bruker	https://brukerspatialbiology.com

### Experimental model and study participant details

#### Mice used in this study

C57BL/6J female mice were purchased from Envigo (France) and were housed in pathogen-free conditions. All animal procedures were in accordance with Swiss legislation on animal experimentation and were approved by the Veterinary Authorities of the Canton Vaud (Authorization VD3308).

#### Cell lines used in this study

The *Trp53*^−/−^, *Trp53*^−/−^*Brca1*^−/−^, and *Trp53*^−/−^*Brca2*^−/−^ ID8 mouse OC cell lines, expressing luciferase as a reporter gene, were derived from spontaneous *in vitro* malignant transformation of C57BL/6 mouse ovarian surface epithelial cells (gift from Prof. Iain A. McNeish, University of Glasgow, Scotland). They closely represent human high-grade serous OC in terms of disseminated peritoneal tumors and ascites fluid formation.^15^^,^^16^ They were maintained at 37°C, 5% CO_2_, in Dulbecco’s Modified Eagle Medium (DMEM) supplemented with 4% fetal bovine serum (FBS), and a combination of 100 μg/mL penicillin, 100 μg/mL streptomycin, ITS (5 mg/mL insulin, 5 mg/mL transferrin, and 5 ng/mL sodium selenite) (All from ThermoFisher), and 400 μg/mL hygromycin (Chemie Brunschwig) to select the cells expressing luciferase reporter. All cell lines were tested and found to be negative for Mycoplasma contamination.

### Method details

#### Murine ovarian cancer *in vivo* model

Eight-week-old C57BL/6J mice were implanted intraperitoneally with 5 x 10^6^ ID8 cancer cells. Animals were weighed at least twice weekly and euthanized when their body weight exceeded 20% as a surrogate endpoint for survival, or when they became distressed and moribund in compliance with the institutional animal care guidelines. For the DSP experiments and T cell analysis, animals were sacrificed 3 weeks after the last OCDC vaccine.

#### OCDC vaccine preparation

Mouse OCDC vaccine preparation was previously described.^43^ Briefly, bone marrow-derived DCs were generated by differentiating bone marrow precursor cells from syngeneic C57BL/6 mice (1-2 × 10^6^ cells/ml) for 5 days in Iscove’s Modified Dulbecco’s Medium (IMDM) containing 10% FBS, 50 μM 2-mercaptoethanol (ThermoFisher), 100 μg/mL penicillin, 100 μg/mL streptomycin, 1000 IU/mL recombinant mouse granulocyte–macrophage colony-stimulating factor (GM-CSF; PeproTech), and 100 IU/mL IL-4 (Peprotech). At day 6, to generate the OCDC vaccine, DCs were pulsed with HOCl-oxidized ID8 tumor lysate prepared as previously described,^43^ at a 1:1 cell ratio. At day 7, DCs were matured with 120 IU/mL lipopolysaccharide (LPS) (Invivogen) and 4000 IU/mL interferon gamma (IFNγ) (PeproTech) for 16 h, then used for vaccination.

#### Mouse treatments

Treatment of mice carrying ID8 tumors started 10 days after tumor graft and continued for 3 weeks, or according to the schedules described in the figures. Mice were vaccinated intradermally with 1 x 10^6^ OCDC every week. Anti-VEGF (40 μg, together with OCDC, Genentech) and anti-PD-1 (200 μg, every 3 days, BioXcell) were given intraperitoneally (i.p.). Olaparib (PARPi) was administered orally at 40 mg/kg/day. As treatment controls, tumor-bearing mice were treated with PBS or isotype controls.

#### Assessment of tumor growth

Tumor burden was monitored by bioluminescence imaging (BLI). BLI was performed before the first treatment and then weekly, until the onset of ascites formation, for each experimental group. Images were acquired using an IVIS Lumina S5 (PerkinElmer) system, and data were analyzed with Living Image 4.7 software (PerkinElmer). Briefly, mice bearing luciferase-expressing ID8 cancer cells were injected i.p. with 150 μg D-luciferin and imaged for 1 min under isoflurane anesthesia.

#### Digital spatial profiling experiment

The Bruker NanoString GeoMx platform was utilized for whole-transcriptome analysis with spatial resolution. Tissue microarrays were prepared by extracting 2 mm punches from FFPE blocks to prepare nine slides, each containing up to 30 tumor sections. Slides were then processed according to the manufacturer’s instructions (Nanostring). Sections were stained with anti-pan-cytokeratin (PanCK)-AF647, anti-CD3-AF488, anti-CD68-AF594, and SYTO 13 dye for nuclei staining (Figure S1). Six hundred forty-eight ROIs were selected, then subdivided into panCK-low, panCK-medium and panCK-high compartments, based on the level of panCK expression. To infer malignant and immune cell composition from spatial transcriptomic data, we performed deconvolution analysis using a single-cell RNA-seq reference dataset derived from mouse ovarian cancer models.^29^ Deconvolution was performed using CIBERSORTx^44^ to estimate proportions of T, B, myeloid, erythroid, stromal, and malignant cells in each ROI (Figure S2). All analyses were performed on PanCK-low, panCK-medium and panCK-high compartments. Then, for tumor cell-enriched compartments, the panCK-high-ROIs were considered, while panCK-low-ROIs were used for immune cell-enriched compartments. Libraries were prepared according to the manufacturer’s instructions and kits, and sequencing was performed on NovaSeq 6000/X.

#### Gene expression signature analyses

Raw counts were exported from GeoMx DSP for downstream analysis in R language for statistical computing. Negative probes were combined by using the geometric mean. ROIs obtained from blood vessels and from control tonsils were removed from the analysis. Additionally, ROIs with low number of reads, low sequencing saturation and low number of genes per ROI were filtered out of the analysis. Two ROIs with very low background were filtered out of the analysis. Next, since GeoMx DSP analysis was run in two different experiments, first from each experiment background was subtracted, then the minimum value was added per experiment, after which the Q3 normalization was performed on the merged experiments.

Gene set enrichment scores were computed using the *GSVA* R package. Heatmaps were generated using the *pheatmap* R package while fold change barplots were implemented with the *ggplot2* R package (Wilcoxin test with Bonferroni correction was used to obtain *p*-values). Hallmark collection of gene sets defining general biological processes was obtained from the MSigDB database.^45^^,^^46^ Immune and T cell subset gene signatures were based on an in-house collection of published gene signatures (Table S1).^18^^,^^19^^,^^20^^,^^21^^,^^22^

#### PD-L1 phenotyping

The *Trp53*^−/−^, *Trp53*^−/−^*Brca1*^−/−^, and *Trp53*^−/−^*Brca2*^−/−^ ID8 mouse cell lines were suspended in DMEM supplemented with FBS, and penicillin/streptomycin and treated with 50 IU/ml of IFNγ and Olaparib (10 μg/mL) for 48h. Cells were then harvested and analyzed by flow cytometry.

#### Monitoring of *ex vivo* T cell responses

Three weeks after the last OCDC vaccine, T cell infiltration and function were monitored using either flow cytometry or IFNγ-ELISpot (Diaclone). Tumors were minced with a razor blade and digested with a mixture of DNase I (25 μg/mL) and Liberase (60 μg/mL) (Roche Diagnostics) in DMEM for 1 h at 37°C, with rotation. Following enzymatic digestion, the samples were passed through a 100 μm filter and washed in PBS containing 2 mM EDTA and 0.5% BSA. Cells were then directly stained for flow cytometry. Ascites (accumulation of fluid in the peritoneal cavity containing tumor cells, immune cells and red cells) were collected and red blood cells were lysed with RBC lysis buffer (Biolegend). Spleens were dissociated to obtain a single-cell suspension. Red blood cells were lysed with RBC lysis buffer. Cells were either directly stained for flow cytometry, or T cells were isolated (Easysep mouse T cells or mouse CD8^+^ T cell isolation kit; Stemcell) and plated for killing assays, IFNγ-ELISpot assay, cytokine production assessment or serial restimulation assay.

##### *In vitro* killing assays

5x10^4^ *Trp53*^−/−^*Brca1*^−/−^ ID8 tumor cells suspended in DMEM supplemented with FBS, and penicillin/streptomycin were plated per well in a 96-well plate. 1 × 10^5^ T cells isolated from OCDC-, OCDC + PARPi-, OCDC + anti-PD-1- or OCDC + PARPi + anti-PD-1-treated mice and suspended in RPMI media supplemented with FBS and penicillin/streptomycin were added. After 16h, supernatant and adherent cells were collected and analyzed by flow cytometry.

##### Cytokine production assessment

Splenocytes were exposed to Cell Activation Cocktail containing brefeldin A (Biolegend). After 5h, the cells were collected and analyzed by flow cytometry.

##### Serial restimulation assays

2 × 10^5^ T cells were exposed to anti-CD3 (5 μg/mL) and anti-CD28 (1 μg/mL) activating antibodies (BD Biosciences) every 3 days 24h after each stimulation, cells were collected and analyzed by flow cytometry.

##### Flow cytometry

Cell suspensions were filtered using a cell strainer (70 μm) and washed in washing buffer (PBS containing 2 mM EDTA and 2% FBS). After incubation with an anti-FcgIII/II receptor antibody (CD16/CD32, Biolegend), live/dead staining was performed using LIVE/DEAD Fixable Blue Dead Cell Stain Kit (ThermoFisher) before subsequent extracellular staining. After incubation, cells were fixed with a fixation & permeabilization solution (Fix & Perm Kit, BD Biosciences). Intracellular staining with anti-TOX, anti-Eomes, anti-IL-2, anti-IFNγ, anti-TNFα and anti-Ki67 antibodies was performed for 30 min on ice. All samples were analyzed using a BD Biosciences Symphony flow cytometer. Compensation was performed using fluorescence minus one (FMO) controls. Cells were discriminated using the following combinations of cell markers after gating on single cells (discriminated by FSC-A and FSC-H) and excluding non-viable cells (LIVE/DEAD negative). T cell infiltration was determined using anti-CD45 (30-F11), anti-CD3 (145-2C11), anti-CD4 (GK1.5), and anti-CD8 (53–6.7) antibodies. T cell exhaustion was determined using anti-CD4 (GK1.5), anti-CD8 (53–6.7), anti-LAG-3 (C9B7W), anti-PD-1 (J43), anti-Tim-3 (RMT3-23), anti-CD39 (Duha59), anti-TIGIT (1G9), anti-TOX (NAN448B) and anti-Eomes (X4-83) antibodies. T cell memory was determined using anti-CD4 (GK1.5), anti-CD8 (53–6.7), anti-CD62L (MEL-14) and anti-CD44 (IM7). T cell polyfunctionality was determined using anti-CD4 (GK1.5), anti-CD8 (53–6.7), anti-IL-2 (JES6-5H4), anti-TNFα (MP6-XT22) and anti-IFNγ (XMG1.2). T cell proliferation was determined using anti-CD3 (145-2C11), anti-41BB (17B5) and anti-Ki67 (16A8). PD-L1 expression was determined using anti-PD-L1 (10F.9G2). For the killing assays co-cultures, the living cells were determined by the population negative for AnnexinV (Biolegend) and DAPI (Sigma). The % of death was calculated as: (Living cells in sample-living cells in control)/living cells in control∗(-100).

##### IFNγ-ELISpot assay

IFNγ-ELISpots assays were performed according to the manufacturer’s instructions (Diaclone). Briefly, B cells isolated from C57BL/6J splenocytes (Easysep mouse B cell isolation kit, Stemcell) and activated for two days with anti-CD40 (5 μg/mL; ThermoFisher) and CpG ODN 1668 (1 mM; Invivogen) were used as antigen-presenting cells (APCs). APCs were pre-pulsed for 24h with HOCl-oxydized ID8 tumor lysate, then cocultured with T cells or CD8^+^ T cells at a ratio of 2 T cells:1 APC (1 × 10^5^ T cells per well) for 15–20 h at 37°C. T cells exposed to phorbol 12-myristate 13-acetate (PMA) and ionomycin (eBioscience) served as positive controls. T cells alone served as background. The IFNɣ spot-forming cells were revealed following the manufacturer’s instructions. The number of IFN-γ-producing cells was counted with the Mabtech ASTOR 2 ELISpot reader.

### Quantification and statistical analysis

Median survival times were calculated using the Kaplan–Meier method and the log rank test. Forest plots for the Cox proportional hazards model were generated using the *survival* R package. FCS files were analyzed by FlowJo software. Statistical computations were performed using R or GraphPad Prism 9 software. All statistical details of the experiments can be found in the figure legends.
