# Supplementary material for: Reversal of T cell exhaustion enables cancer vaccine efficacy in BRCA1-deficient ovarian cancer
Source: iScience. 2026 Jun 30;29(7):116587. doi: 10.1016/j.isci.2026.116587 (PMC13378375; doi:10.1016/j.isci.2026.116587)
Supplement: Document S1. Figures S1–S11 and Table S1 [file mmc1.pdf]

iScience, Volume *29*

Supplemental information

Reversal of T cell exhaustion enables cancer vaccine efficacy in *BRCA1*-deficient ovarian cancer

Laurent Beziaud, Aspram Minasyan, Cheryl L-L Chiang, Rania M. Soukarieh, Matilde M. Coppi, Raphaël Rovelli, Jonathan Thevenet, Stephanie Tissot, Denarda Dangaj-Laniti, and Lana E. Kandalaft

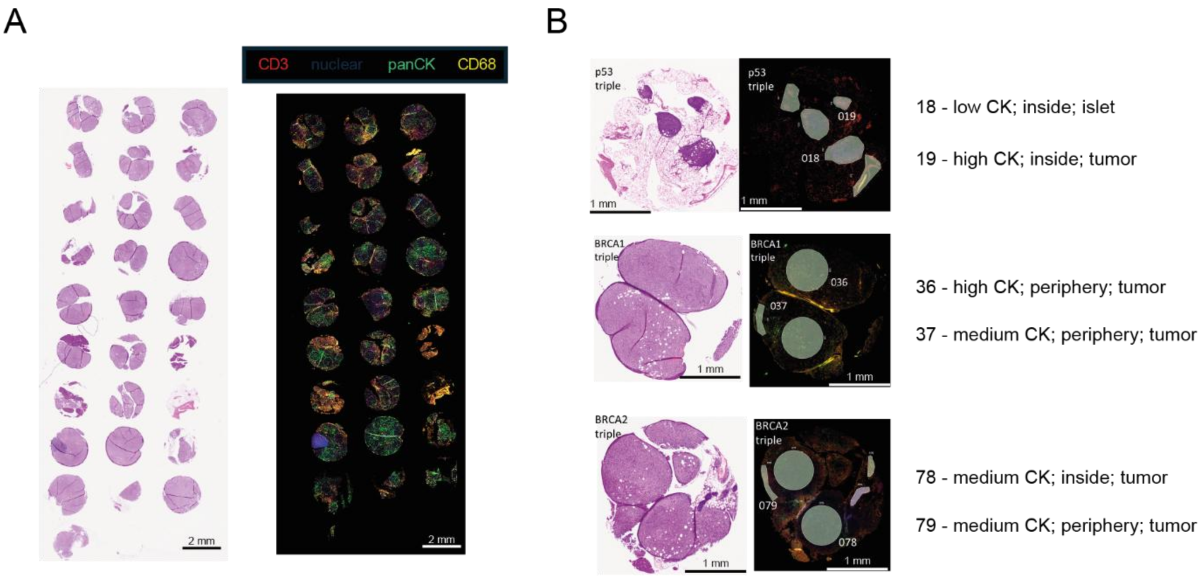

Supplemental figure S1. Representative images of data acquisition using the GeoMx digital spatial profiling platform. (A) Tissue microarrays were prepared by extracting 2 mm punches from FFPE blocks, and sections were stained with anti-pan-cytokeratin (PanCK), anti-CD3, anti-CD68, and SYTO 13 dye. (B) Regions on the GeoMx scans were chosen based on the location of tumors on the H&E stains and the immunostaining patterns of Pan-CK.

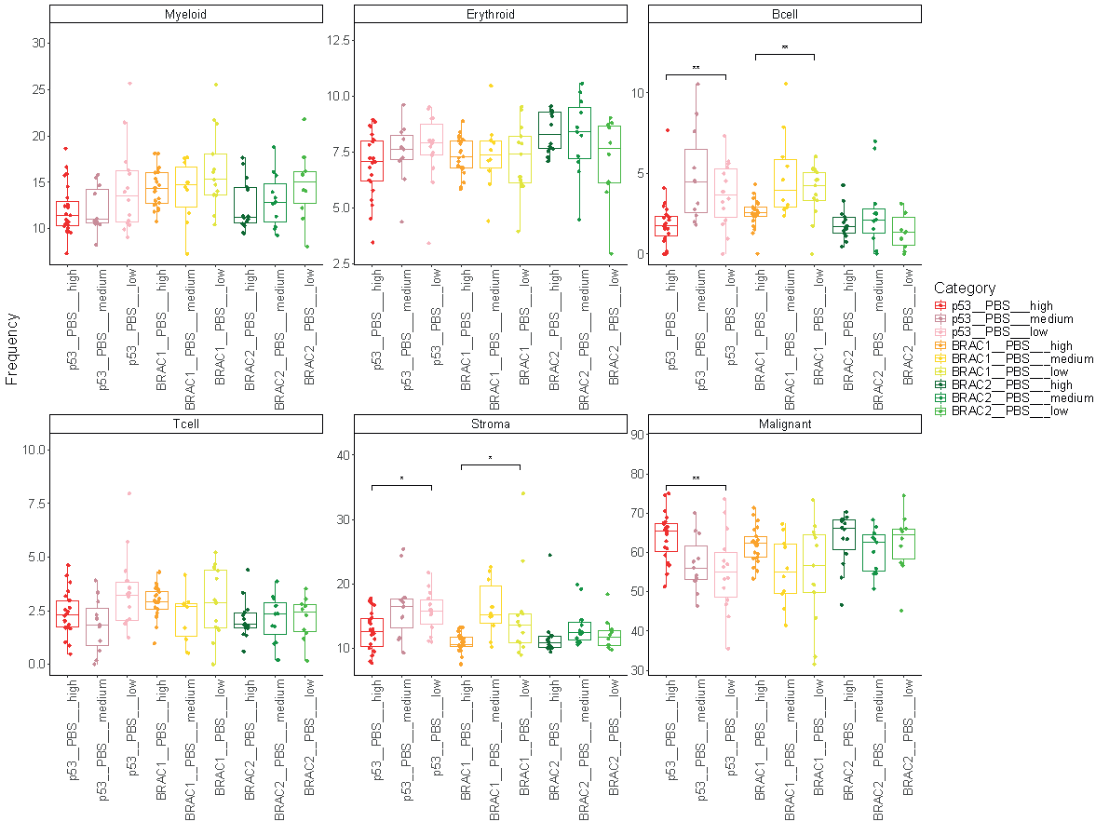

Supplemental figure S2. Immune and malignant cells frequency according to pan-cytokeratin expression levels. Regions on the GeoMx scans were subdivided in pan-cytokeratin (PanCK)-low, panCK-medium and panCK-high compartments, based on the level of panCK expression. Myeloid, erythroid, B, T, stromal and malignant cells frequency were inferred in the various panCK-expressing compartments. Representative graphs from the PBS-treated samples in the *Trp53*$^{-/-}$, *Trp53*$^{-/-}$*Brca1*$^{-/-}$ and *Trp53*$^{-/-}$*Brca2*$^{-/-}$ are shown; boxplots defined as the box (median and first and third quartiles) and whisker (extreme value). *p < 0.05, **p < 0.01.

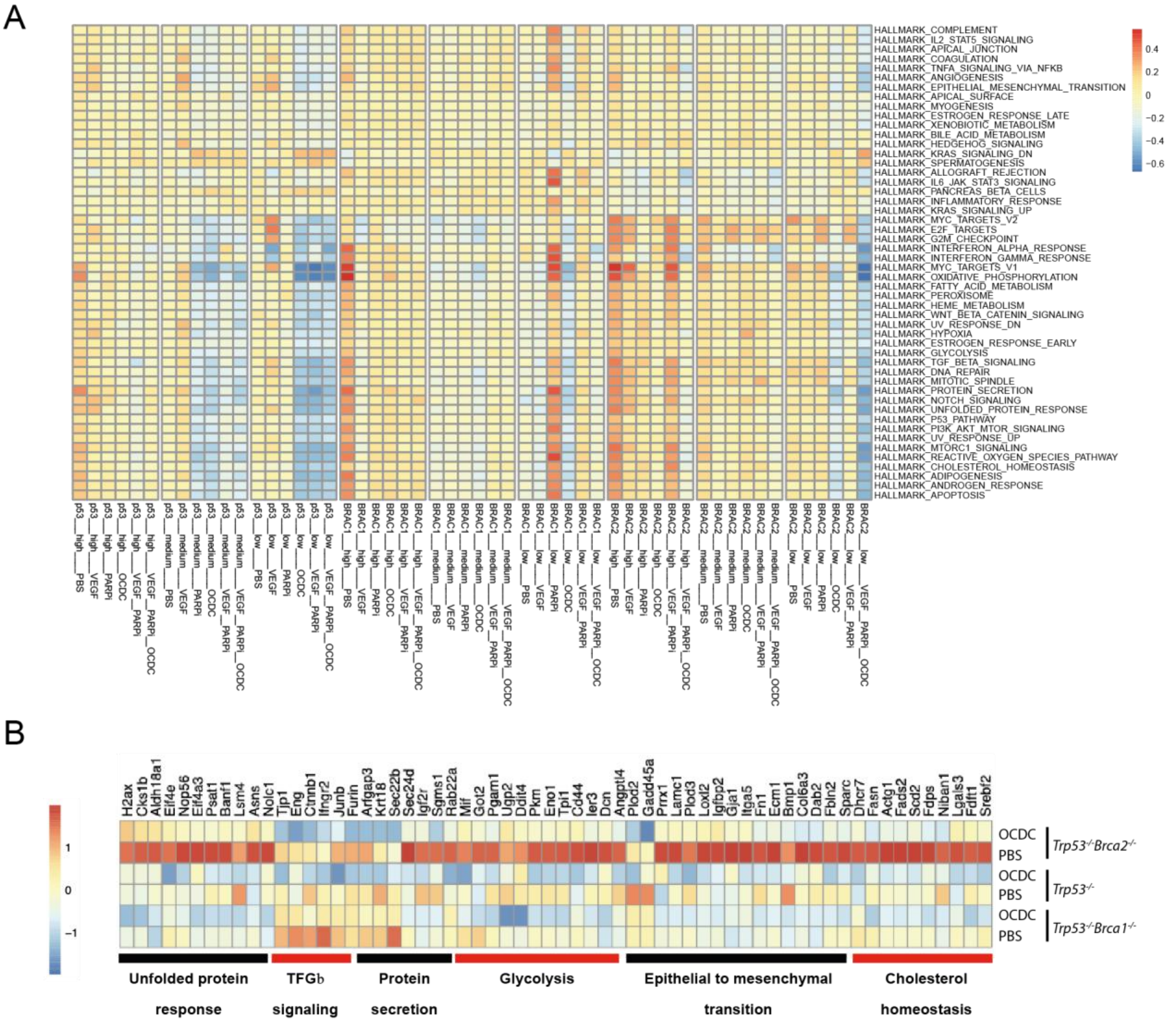

Supplemental figure S3. Heatmaps of hallmark gene signatures of general biological processes. (A) Heatmap of hallmark gene signatures of general biological processes in the various panCK-expressing compartments of different treatment groups (PBS; anti-VEGF; PARPi; OCDC; anti-VEGF + PARPi; anti-VEGF + PARPi + OCDC). Scale bar represents median GSVA enrichment score. (B) Heatmap of genes composing general biological processes in the tumor cell-enriched (panCK high) compartments of OCDC and PBS groups, in the *Trp53*$^{-/-}$, *Trp53*$^{-/-}$*Brca1*$^{-/-}$, and *Trp53*$^{-/-}$*Brca2*$^{-/-}$ ID8 tumor models. Scale bar represents median expression score.

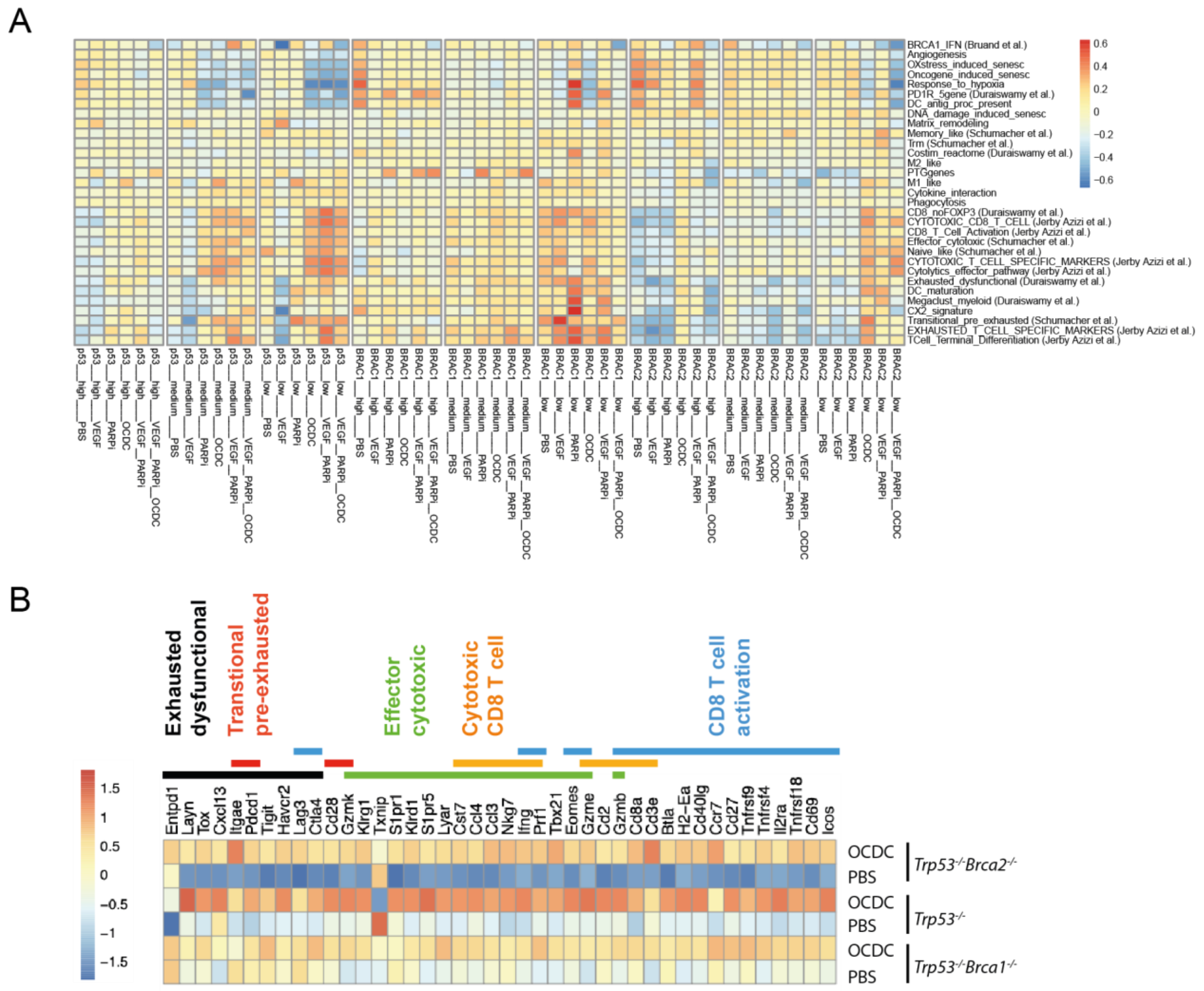

Supplemental figure S4. Heatmap of immune and T cell subsets gene signatures. (A) Heatmap of immune and T cell subsets gene signatures in the various panCK-expressing compartments of different treatment groups (PBS; anti-VEGF; PARPi; OCDC; anti-VEGF + PARPi; anti-VEGF + PARPi + OCDC). Scale bar represents mean GSVA enrichment score. (B) Heatmap of genes from exhaustion and activation T cell signatures in the immune cell-enriched (panCK low) compartments of OCDC and PBS groups, in the *Trp53*$^{-/-}$, *Trp53*$^{-/-}$*Brca1*$^{-/-}$, and *Trp53*$^{-/-}$*Brca2*$^{-/-}$ ID8 tumor models. Scale bar represents mean expression score.

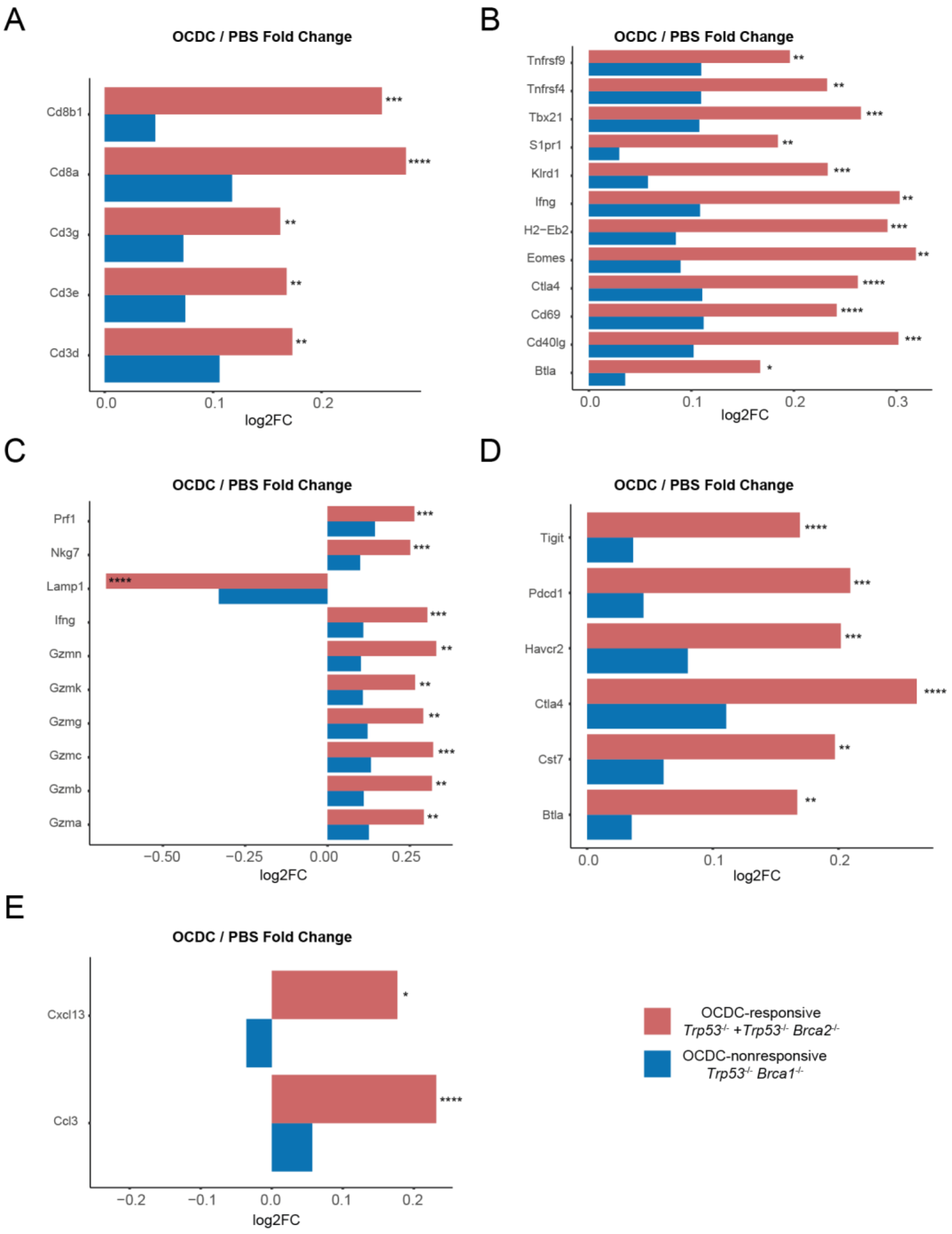

Supplemental figure S5. Fold changes of immune genes between the OCDC and PBS groups. Bar plots displaying the log2 fold change of T cell subset genes in immune cell-enriched compartments, between OCDC and PBS groups in the OCDC-responsive (*Trp53*$^{-/-}$ and *Trp53*$^{-/-}$*Brca2*$^{-/-}$) and in the OCDC-nonresponsive (*Trp53*$^{-/-}$*Brca1*$^{-/-}$) ID8 tumor models (Wilcoxin test with Bonferroni correction). (A) T cell markers. (B) Costimulatory and activation markers. (C) Cytotoxic effector molecules. (D) Immune checkpoint and inhibitory receptors. (E) Chemokines. *$p < 0.05$, **$p < 0.01$, ***$p < 0.001$, ****$p < 0.0001$.

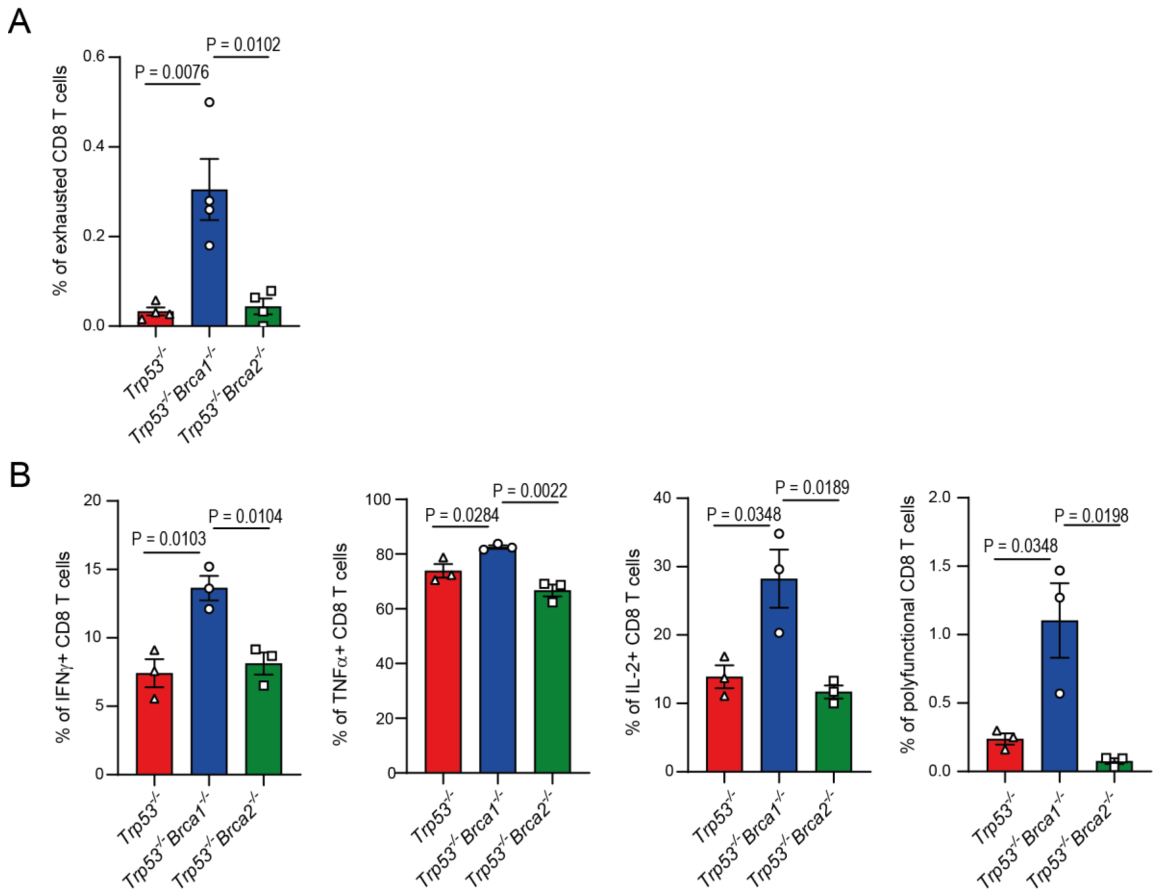

Supplemental figure S6. Increased level of exhausted and polyfunctional CD8 T cells in the spleen of mice implanted with *Trp53$^{-/-}$Brca1$^{-/-}$* ID8 tumor model. Quantification of (A) PD-1^{hi}Tim-3^{+}Lag-3^{+}TIGIT^{+}CD39^{+}Eomes^{+}TOX^{hi} exhausted CD8 T cells and (B) IFNγ-, TNFα-, or IL-2-producing CD8 T cells and polyfunctional IFNγ^{+}TNFα^{+} IL-2^{+} CD8 T cells infiltrated in spleen of *Trp53$^{-/-}$*, *Trp53$^{-/-}$Brca1$^{-/-}$*, and *Trp53$^{-/-}$Brca2$^{-/-}$* ID8-bearing mice by flow cytometry (n = 3; graphs representative of 2 individual experiments; data are represented as mean +/- SEM; unpaired parametric t test).

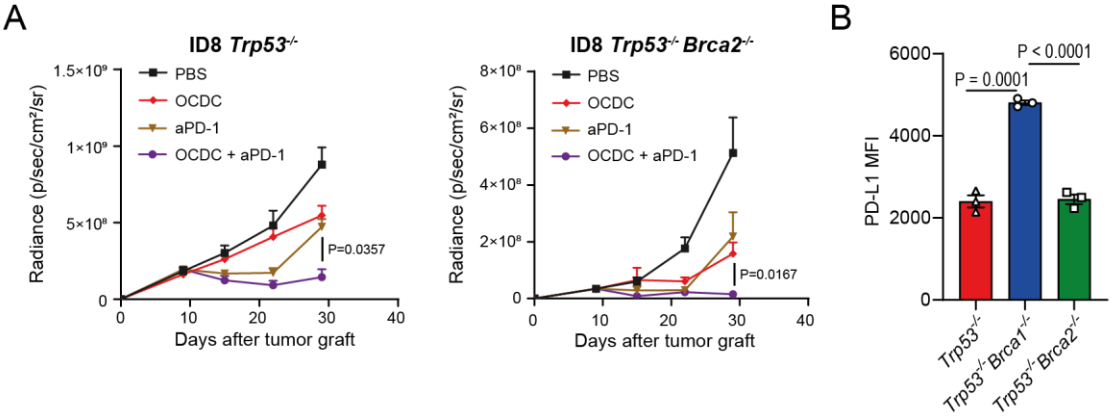

Supplemental figure S7. Anti-PD-1 improves the anti-tumor efficacy of OCDC vaccine in the *p53* and *Brca2*-deficient ID8 tumor model. C57BL/6J mice were injected intraperitoneally with *Trp53*$^{-/-}$ or *Trp53*$^{-/-}$*Brca2*$^{-/-}$ ID8 tumors, then treated with OCDC vaccine and anti-PD-1. (A) Tumor growth kinetics by bioluminescence measurements (n = 3 mice per group; data are represented as mean +/- SEM; unpaired nonparametric Mann-Whitney test). (B) Quantification of the mean fluorescence intensity (MFI) of PD-L1 expression on *Trp53*$^{-/-}$, *Trp53*$^{-/-}$*Brca1*$^{-/-}$ or *Trp53*$^{-/-}$*Brca2*$^{-/-}$ ID8 cell lines *in vitro* by flow cytometry (n = 3; graphs representative of 2 individual experiments; data are represented as mean +/- SEM; unpaired parametric t test).

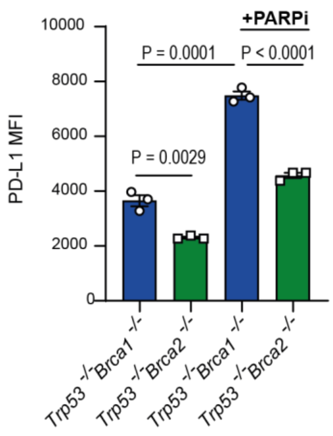

Supplemental figure S8. PARPi increases PD-L1 expression in ID8 tumor models. Quantification of PD-L1 expression on *Brca1*- and *Brca2*-deficient ID8 tumor cell lines treated *in vitro* with PARP inhibitor by flow cytometry (n = 3; graph representative of 2 individual experiments; data are represented as mean +/- SEM; unpaired parametric t test).

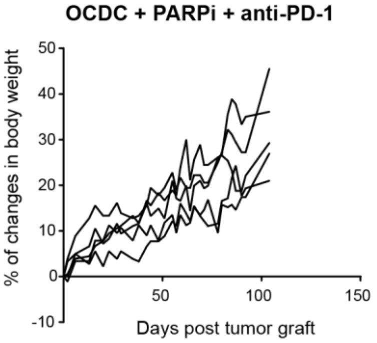

Supplemental figure S9. No body weight loss following anti-PD-1, PARPi and OCDC vaccine triplet therapy. C57BL/6J mice were injected intraperitoneally with *Trp53*$^{-/-}$ *Brca1*$^{-/-}$ ID8 tumors and treated with OCDC vaccine, PARPi and anti-PD-1. Longitudinal changes in body weight compared to the day of tumor graft are shown per mouse (n = 5 mice; graph representative of 5 individual experiments).

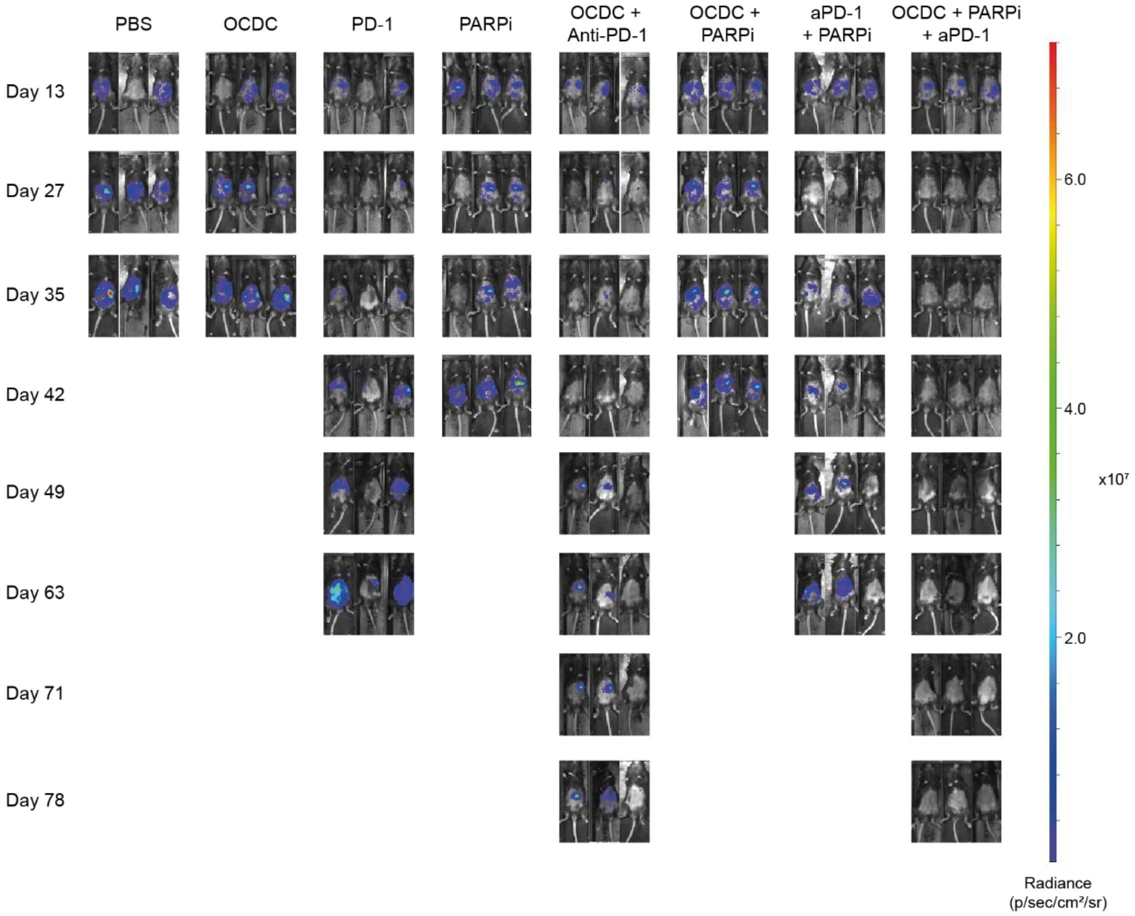

Supplemental figure S10. Anti-PD-1, PARPi and OCDC vaccine triplet therapy promotes durable tumor control. C57BL/6J mice were injected intraperitoneally with *Trp53*$^{-/-}$ *Brca1*$^{-/-}$ ID8 tumors and treated with OCDC vaccine, PARPi and anti-PD-1. Representative bioluminescence (BLI) measurements.

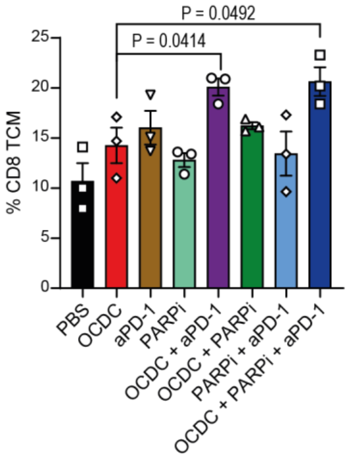

Supplemental figure S11. Anti-PD-1, PARPi and OCDC vaccine triplet therapy increases the pool of memory CD8 T cells in *Brca1*-deficient ID8 tumor model. C57BL/6J mice injected intraperitoneally with *Trp53*$^{-/-}$*Brca1*$^{-/-}$ ID8 tumors were treated with OCDC vaccine, PARPi or anti-PD-1. Quantification of CD44^{hi}CD62L^{+} central memory (TCM) CD8 T cells by flow cytometry in the splenocytes (n = 3; graph representative of 2 individual experiments; unpaired parametric t test).

Gene signatures	Genes
Exhausted_dysfunctional (Duraiswamy et al.)	Tigit, Lag3, Entpd1, Layn, Tox, Havcr2, Cxcl13, Ctla4, Pdcd1, Itgae
Costim_reactome (Duraiswamy et al.)	Ppp2r5c, Prr5, Btla, Rictor, Trbc1, Trbc2, Cd80, Trbv15, Trbv16, Pik3r1, H2-Eb2, Tnfrsf10b, Cd274, Pdcd1lg2, H2-Ab1, Pdpk1, Ppp2r5b, H2-Ea, Mlst8, Mtor, Fyn, Trav16d-dv11, Trav16n, Ppp2r5e, Map3k8, Lck, Ppp2r1b, Grb2, Ptpn6, Cd4, Vav1, Akt3, Ppp2cb, Cd3g, Ppp2r1a, Ctla4, Akt2, Cd3e, Cd28, Tnfrsf14, Ppp2r5a, Trav16, Grap2, Ppp2r5d, Icos, Pak3, Ppp2ca, Trav19, Pik3ca, Csk, Cd247, Cdc42, Cd86, Cd3d, Gm49337, Pik3r3, Pik3r2, Them4, Akt1, Pak1, Pak2, Trib3, Ptpn11, Map3k14, Lyn, Icosl, Yes1, Mapkap1, Rac1, Pdcd1, Src
CD8_noFOXP3 (Duraiswamy et al.)	Tigit, Cd27, Lag3, Cd69, Gzme, Gzmd, Gzmg, Gzmn, Gzmf, Gzmc, Gzmb, Havcr2, Il15ra, Prf1, Il2ra, Itga4, Pdcd1, Cd244a, Itgae
PD1R_5gene (Duraiswamy et al.)	Cd74, H2-Eb2, Clic1, Cxcl13
Megaclust_myeloid (Duraiswamy et al.)	Cd80, Csf1r, Cd14, H2-Eb2, H2-Ea, Cd274, Nectin2, Cd86, Itgam, Gm49368, Cd40, Ptprc
DNA_damage_induced_senesc	Lmnb1, Kat5, Pot1b, Ccna2, Acd, Terf2, Ubn1, Rb1, Hmga2, Atm, Hira, Ep400, Asf1a, Cabin1, Terf2ip, Cdkn1b, Pot1a, Cdk2, Ccna1, Hmga1b, Rad50, Mre11a, Terf1, Tinf2, Cdkn1a, Trp53, Ccne2, Ccne1, Nbn
OXstress_induced_senesc	Fos, Mapk1, Phc1, Mdm2, Ezh2, Mapk11, Cdk6, E2f3, Mapk10, Rbbp7, Tfdp1, Cdk4, Rbbp4, Mapkapk3, E2f1, Map2k7, Gm49320, Map2k3, Bmi1, Cbx4, Ago3, E2f2, Mapk8, Ubc, Suz12, Ifnb1, Tnik, Map2k4, Mapk14, Cbx8, Uba52, Eed, Ago4, Ring1, Mov10, Mapkapk2, Kdm6b, Ubb, Cbx6, Tnrc6b, Mdm4, Cbx2, Mapk9, Tnrc6c, Map3k5, Ago1, Phc2, Scmh1, Jun, Cdkn2b, Cdkn2c, Tfdp2, Mapk3, Phc3, Txn1, Mink1, Cdkn2d, Map4k4, Map2k6, Rnf2, Tnrc6a, Gm42878, Mapkapk5, Trp53
Oncogene_induced_senesc	Mapk1, Ets2, Mdm2, Rb1, Cdk4, Tfdp1, Ets1, Cdk6, E2f3, Ubc, Cdkn2c, Uba52, Mov10, Sp1, Ago4, Tfdp2, Tnrc6b, Mapk3, Ubb, Mdm4, E2f1, Tnrc6c, Ago1, Id1, E2f2, Cdkn2b, Ago3, Trp53, Tnrc6a, Cdkn2d, Erf
Response_to_hypoxia	Egln3, Psma6, Psmb10, Psmc1, Psmd4, Epas1, Psmb9, Psmb4, Ube2d2a, Psmb8, Psmb3, Hif1a, Psma3, Crebbp, Psmd2, Egln2, Psma8, Hif1an, Cul2, Psma4, Psmd7, Arnt, Psmd3, Ubb, Ube2d1, Psmb1, Psma5, Psmc2, Cited2, Uba52, Higd1a, Psmd11, Rbx1, Ubc, Psmd6, Psmb11, Psmd10, Psma7, Psmd8, Psma1, Psme3, Vhl, Vegfa, Psmb5, Psmb2, Ajuba, Sem1, Psmc6, Eloc, Hif3a, Ep300, Psme4, Psmc3, Psme1, Psma2, Psmc4, Psme2, Psme2b, Psmd9, Psmf1, Psmc5, Psmb7, Psmd5, Elobl, Psmd14, Limd1, Psmb6, Car9, Psmd12, Epo, Psmd13, Wtip, Psmd1
PTGgenes	Ptger4, Ptger2, Ptges, Ptgs2, Ptgs1
CYTOTOXIC_CD8_T_CELL (Jerby Azizi et al.)	Cd8b1, Cd8a, Ifng, Nkg7, Gzme, Gzmd, Gzmg, Gzmn, Gzmf, Gzmc, Gzmb, Gzma, Cd3g, Cd2, Prf1, Cd3e, Cd3d, Ccl4, Cst7, Ccl3
CYTOTOXIC_T_CELL_SPECIFIC_MARKERS (Jerby Azizi et al.)	Ifng, Nkg7, Gzme, Gzmd, Gzmg, Gzmn, Gzmf, Gzmc, Gzmb, Gzma, Prf1, Cst7, Ccl3, Ccl4
EXHAUSTED_T_CELL_SPECIFIC_MARKERS (Jerby Azizi et al.)	Tigit, Lag3, Havcr2, Ctla4, Pdcd1
CD8_T_Cell_Activation (Jerby Azizi et al.)	Btla, Ifng, Cd8a, Cd47, Cd8b1, H2-Ea, Cd40lg, Ccr7, Lamp1, Cd27, Tnfrsf9, Lag3, Tnfrsf4, Il2ra, Tnfrsf18, Gzma, Gzme, Gzmd, Gzmg, Gzmn, Gzmf, Gzmc, Gzmb, Eomes, Cd69, Ctla4, Cd3e, Prf1, Icos
Cytolytics_effector_pathway (Jerby Azizi et al.)	Gzme, Gzmd, Gzmg, Gzmn, Gzmf, Gzmc, Gzmb, Eomes, Gzma, Prf1, Tbx21
TCell_Terminal_Differentiation (Jerby Azizi et al.)	Tigit, Cd274, Lag3, Cd160, Havcr2, Ctla4, Cd244a, Pdcd1
BRCA1_IFN (Bruand et al.)	Ifi44l, Ifi44, Ifit2, Mx1, Mx2, Isg20, Stat1, Dhx58, Isg15, Ifih1, Cxcl10, Oasl1, Irf7, Zbp1, Ddx58
CX2_signature (Bruand et al.)	Cd8a, Laptm5, Cd4, Tnfrsf9, Lcp1, Il10ra, Cd86, Gbp2b, Gbp2, Stat1, Cd3e, Slc7a7, Gzmk, Cd2, Cxcl9, Cd53, Cd247, Irf1, Il2rb, Dock2, Ptprc
Naive_like (Schumacher et al.)	Il7r, Lef1, Ccr7, Cd27, Tcf7, S1pr1, Sell, Cd28
Memory_like (Schumacher et al.)	Il7r, Gpr183, Lef1, Ccr7, Cd27, Ltb, Ankrd28, Myadm, Cd28, S1pr1, Gzmk, Cxcr5, Eomes, Sell, Vim, Tcf7, Atp2b1, Gzma
Effector_cytotoxic (Schumacher et al.)	Klrg1, Ifng, Nkg7, Txnip, S1pr1, Gzmk, Klrd1, Gzma, Prf1, Eomes, Cx3cr1, Gzme, Gzmd, Gzmg, Gzmn, Gzmf, Gzmc, Gzmb, S1pr5, Fcgr4, Lyar, Ccl3, Tbx21, Cst7, Gzmm, Ccl4
Transitional_pre_exhausted (Schumacher et al.)	Cd28, Gzmk, Pdcd1, Itgae
Trm (Schumacher et al.)	Tigit, Ccna2, Ifng, Cd6, Capg, Top2a, Hmgb2, Lag3, Nr4a1, Tnfrsf9, Entpd1, Tuba1b, Cks1b, Xcl1, Havcr2, Cxcl13, Cd69, Stmn1, Tubb5, Rora, Gzma, Gzme, Gzmd, Gzmg, Gzmn, Gzmf, Gzmc, Gzmb, Kif2c, Myadm, Ctla4, Prf1, Itgae, Mki67, Ccl3, Nr4a2, Pdcd1, Nr4a3
M1_like	Il12a, Tnf, Cmklr1, Irf5, Il12b, Socs3, Nos2, Il23a, Il1b

M2_like	Csf1r, Ctsb, Tgfb3, Wnt7b, Cd163, Vegfc, Irf4, Tgfb1, Tnfsf8, Egf, Ccl20, Vegfa, Il10, Ccl17, Mmp14, Ccl22, Vtcn1, Vegfd, Lyve1, Clec7a, Msr1, Cd276, Ctsc, Vegfb, Mrc1, Mmp19, Tgfb2, Il4i1, Gm49369, Ctsd, Ccl3, Tnfsf12, Il4ra, Mmp9, Ctsa, Siglec1, Fn1, Ccl4, Ccl24
Angiogenesis	Edn1, Tymp, Ptk2, Stc1, Ezh2, E2f3, Fyn, Ccnd2, Spp1, Jag2, Vegfa, Cxcr4, Vcan, Hey1, Fgf18, Vav2, Fgfr1, Mmp9, Cd44, Tnfaip6, Ccne1, Notch1, Jag1, Itgav, Pdgfa
Cytokine_interaction	Il13ra1, Tnfrsf13c, Csf1r, Ifnar1, Il7r, Il12rb2, Il17b, Tslp, Tgfb3, Il23r, Ifngr2, Prlr, Pdgfrb, Il12a, Lifr, Gm21970, Il10rb, Osmr, Il17rb, Ifnar2, Tnfrsf19, Prl, Prl3d1, Prl3d2, Prl3d3, Prl3c1, Prl3b1, Prl3a1, Prl6a1, Prl8a2, Prl2b1, Prl8a6, Prl8a8, Prl8a9, Prl8a1, Prl7b1, Prl7a1, Prl7a2, Prl7d1, Prl7c1, Prl2a1, Prl2c1, Prl4a1, Prl5a1, Fas, Csf2ra, Tnfrsf10b, Pdgfc, Ifng, Tpo, Il22, Il22b, Lta, Il21, Cd70, Vegfc, Tnfsf8, Csf2rb2, Csf2rb, Acvrl1, Acvr1b, Tnfsf9, Il18, Tnfrsf1a, Tnfrsf17, Il15, Tnf, Il2, Tnfsf14, Csf3, Cd40lg, Ccr7, Met, Ltbr, Tnfrsf11b, Il1rap, Inhbb, Ccl20, Il6st, Tgfb1, Ltb, Tnfsf11, Tnfrsf9, Egf, Cd27, Tnfrsf13b, Cxcl13, Plekho2, Il3, Ccr6, Tnfrsf18, Cxcl5, Epor, Tnfsf13b, Xcl1, Cxcr3, Bmpr1b, Tnfrsf1b, Lepr, Il5ra, Inhbe, Cxcl11, Kit, Cxcl14, Ccl28, Flt3l, Il2rb, Pdgfra, Ifne, Ifna15, Ifna14, Ifna9, Ifna12, Ifna13, Ifna16, Ifna2, Ifnab, Ifna7, Ifna11, Ifna6, Ifna5, Ifna4, Ifna1, Mpl, Csf2, Ccl2, Ccl8, Ifnb1, Ccl22, Flt3, Cxcl10, Il15ra, Cx3cr1, Kitl, Il17a, Fasl, Il23a, Il20, Il2ra, Il25, Tnfrsf4, Il5, Ifnl2, Ifnl3, Cxcl12, Csf1, Tnfsf4, Tnfsf10, Cxcr5, Tnfrsf21, Vegfb, Amhr2, Acvr2b, Tgfb2, Tnfrsf8, Il6, Crlf2, Kdr, Flt4, Il11, Lep, Flt1, Ccr4, Cxcr4, Il7, Inhba, Ccl11, Bmpr1a, Vegfa, Il9r, Il24, Cx3cl1, Il17ra, Ctf1, Cxcl9, Edar, Il20rb, Eda2r, Il12rb1, Il19, Prl2c3, Prl2c2, Prl2c5, Il20ra, Ccr8, Il22ra1, Il10ra, Il12b, Ghr, Il9, Ccr10, Il13, Pdgfb, Gdf5, Ifngr1, Ifnlr1, Il10, Ppbp, Cntf, Ccl17, Ccl12, Bmpr2, Il22ra2, Il6ra, Tnfrsf25, Tnfrsf14, Tnfsf18, Hgf, Il4, Clcf1, Inhbc, Csf3r, Ccl7, Gm20489, Il2rg, Eda, Vegfd, Ccl19, Ccl21d, Gm13304, Ccl21b, Gm10591, Ccl21a, Osm, Epo, Il18rap, Ccr1, Ccr1l1, Ccl9, Ccl6, Ccl4, Cntfr, Tnfrsf26, Tnfrsf22, Tnfrsf23, Ccr5, Xcr1, Tnfrsf11a, Il1a, Tnfsf13, Il1r1, Il4ra, Lif, Amh, Ccl3, Tnfsf12, Cd40, Ngfr, Gh, Tgfbr1, Il11ra1, Gm13305, Il11ra2, Ifnk, Cxcr2, Cxcr1, Ccr9, Egfr, Pdgfa, Bmp7, Relt, Ccr2, Ccr3, Cxcr6, Il1r2, Il18r1, Gm13306, Ccl27b, Ccl27a, Acvr2a, Bmp2, Acvr1, Ccl24, Tgfbr2, Cxcl16, Tnfsf15, Il21r, Il1b
Phagocytosis	Megf10, Pgbd1, Scarf2, Scara3, Loxl2, Scara5, Prss12, Cd163, Stab1, Loxl4, Enpp2, Endou, Tmprss2, Ager, Cd5l, Stab2, Tmprss5, Hhipl1, Scarf1, Cfi, Cd5, Tmprss15, Cd6, Tmprss3, Tmprss13, Colec12, Tmprss4, Enpp1, Msr1, Ackr2, Ssc5d, Loxl3, Scarb2, Ackr3, Ackr4, Lgals3bp, Lrp1, Vtn, Cd36, Scarb1, Cxcl16, Prg4, Scart1, Dmbt1, Marco, Ssc4d
Matrix_remodeling	Col1a2, Lamc2, Lama3, Adamts5, Mmp7, Lox, Tnc, Col3a1, Mmp11, Col4a1, Vtn, Lgals7, Col11a1, Plod2, Mmp2, Col5a1, Lamb3, Eln, Fn1, Mmp12, Adamts4, Col1a1, Mmp9, Mmp1a, Mmp1b, Mmp3, Car9
DC_antig_proc_present	Dync1h1, Cd74, Dctn4, Klc2, Kif26a, Bcap31, Ctsf, Klc1, Psma6, Sec23a, BC048507, H2-Ab1, Erap1, Psmc1, H2-DMb2, H2-DMb1, Hfe, Sec24d, Psmb10, Tap2, H2-Ea, Psmb9, H2-Ob, Psmb8, Tap1, Trem2, H2-Eb2, Clec4a1, Rab7, Psmb4, H2-DMa, Psmd4, Fcgr1, H2-D1, H2-Q1, H2-Q2, H2-Q4, H2-Q6, H2-Q7, H2-Q10, Psmd2, Psma3, Psma4, Psmb3, Arf1, Psmd7, Ap1g1, Ap1s2, Ncf2, Lgmn, Calr, Rilp, Ncf4, Racgap1, Ctss, Kif5a, Lag3, Psmd3, Ap2m1, Psma8, Ap1s3, Kif2a, Mr1, Capza2, Ikbkg, Sec24b, Chuk, Sec24c, Tapbpl, Actr10, Kif3c, Dctn2, Osbpl1a, Psma7, Psmd6, Cenpe, Itgb5, Psma1, Psmd8, Psmb1, Psma5, Snap23, B2m, Psmc3, Psmc4, Psmb5, Kif3a, Kif2c, Tapbp, Psmc6, Sptbn2, Vamp8, Kif15, Kif4, 4933400A11Rik, Capza1, Sh3gl2, Psmc2, Vamp3, Cd209b, Cd209c, Cd209a, Cd209e, Cd209d, Kif11, Ikbkb, Psme4, Psmd9, Sec13, Psme1, Dync1i1, Ap2s1, H2-Oa, Marchf1, Dynll1, Capza3, Kif3b, Actr1a, Cybb, Psmd10, Psmb11, Lnpep, H2-T24, H2-T23, H2-T22, Gm11127, Gm7030, H2-T10, Gm8909, H2-T3, H2-M10.2, H2-M10.1, H2-M10.3, H2-M10.4, H2-M11, H2-M9, H2-M1, H2-M10.5, H2-M10.6, H2-M3, H2-M2, H2-M5, H2-K1, Actr1b, Capzb, Dync1i2, Sar1b, Psmd11, Ap1m1, Kifap3, Psme2, Psme2b, Cyba, Psma2, Dync1li2, Pdia3, Cd36, Psmb2, Canx, Sec22b, Dync1li1, Kif22, Psme3, Sec24a, Dctn6, Ap2a1, Ctse, Cd207, Dctn1, Pycard, Cd209f, Cd209g, Ifi30, Psmd13, Clta, Dctn5, Traf6, Ap2b1, Psmf1, Dctn3, Psmd1, Psmb7, Cltc, Ap1s1, Psmc5, Psmd12, Sec31a, Fcgr2b, Fcgr3, Gm49369, Ctsd, Dynll2, Ap1m2, Slc11a1, Ap1b1, Dnm2, Fcer1g, Ace, Psmb6, Kif18a, Psmd5, Psmd14, Kif2b, Ap2a2, Itgav, Ncf1
DC_maturation	Cd80, Cd83, Cd86, Relb, Cd40

Supplemental table S1. Immune cell subset gene signatures. List of genes composing the immune cell and T cell subsets gene signatures, based on an in-house collection of published gene signatures.[18–22]
